# Gasdermin-Mediated Pyroptosis in Hidradenitis Suppurativa: Molecular Insights and Therapeutic Implications

**DOI:** 10.3390/biology14091258

**Published:** 2025-09-12

**Authors:** Kinga Tyczyńska, Piotr K. Krajewski, Aleksandra Sójka, Danuta Nowicka-Suszko, Iwona Bednarz-Misa, Mariusz Fleszar, Małgorzata Krzystek-Korpacka, Jacek C. Szepietowski

**Affiliations:** 1 Clinical Department of Rheumatology and Internal Medicine, Wroclaw Medical University, 50-556 Wroclaw, Poland; kinga.tyczynska@student.umw.edu.pl; 2 Division of Dermatology, Venereology and Clincial Immunology, Faculty of Medicine, Wroclaw University of Science and Technology, 50-377 Wroclaw, Poland; jacek.szpepietowski@pwr.edu.pl; 3 University Centre of General Dermatology and Oncodermatology, Wroclaw Medical University, 50-556 Wroclaw, Poland; aleksandraa.sojka@gmail.com (A.S.); danuta.nowicka-suszko@umw.edu.pl (D.N.-S.); 4 Department of Biochemistry and Immunochemistry, Wroclaw Medical University, 50-368 Wroclaw, Poland; iwona.bednarz-misa@umw.edu.pl (I.B.-M.); malgorzata.krzystek-korpacka@umw.edu.pl (M.K.-K.); 5 Omics Research Center, Wroclaw Medical University, 50-368 Wroclaw, Poland; mariusz.fleszar@umw.edu.pl; 6 Department of Dermato-Venereology, 4th Military Hospital, 50-981 Wroclaw, Poland

**Keywords:** hidradenitis suppurativa, pyroptosis, Gasdermin D, Gasdermin E, inflammation, cytokines, interleukin-1β, interleukin-18, skin disease, biomarkers

## Abstract

Hidradenitis suppurativa (HS) is a debilitating inflammatory skin condition with unclear underlying mechanisms. Recent studies suggest pyroptosis, a highly inflammatory form of programmed cell death, could significantly contribute to the disease process. This study aimed to investigate the roles of gasdermin D (GSDMD) and gasdermin E (GSDME), two key mediators of pyroptosis, in HS pathogenesis. Serum and skin samples from HS patients and healthy individuals were analyzed. Serum gasdermin D levels were significantly elevated in HS patients, although gasdermin E showed no significant difference compared to controls. Skin tissue analysis revealed increased expression of both GSDMD and GSDME in inflamed HS lesions compared to unaffected skin from the same patients and healthy control skin. However, these molecules did not correlate directly with disease severity. Interestingly, systemic metabolic markers showed some associations with gasdermin expression, suggesting potential connections between systemic health and inflammation in HS. These findings confirm a critical role of gasdermins in HS-related inflammation and identify pyroptosis as a promising therapeutic target. Further exploration of these pathways could yield valuable treatment strategies for managing this challenging skin condition.

## Simple Summary

Hidradenitis suppurativa is a chronic skin disease characterized by painful, recurrent skin abscesses and tunnels in areas such as the armpits and groin. It severely impacts patients’ lives, causing discomfort and psychological distress. Despite affecting many people, the exact cause of this condition is not fully understood, making it difficult to manage effectively. Recent studies suggest that a specific type of cell death called pyroptosis, which triggers inflammation, may be involved. This study explored two molecules, gasdermin D and gasdermin E, that play key roles in pyroptosis. The results showed higher levels of these molecules in the affected skin areas of patients compared to healthy skin, indicating their role in promoting inflammation. This discovery is significant because it suggests that reducing the activity of these molecules could help control inflammation in hidradenitis suppurativa. These findings offer new insight into the disease and may lead to improved treatments, providing better quality of life for those affected by hidradenitis suppurativa.

## 1. Introduction

Hidradenitis suppurativa (HS) is a chronic, recurrent inflammatory dermatosis primarily localized in the intertriginous regions such as the axillae, groin, and perineum [1]. The clinical manifestations include painful nodules, abscesses, tunnels, and progressive scarring, which severely impact patients’ quality of life [1,2,3]. While the exact pathogenesis of HS remains elusive, the overproduction of proinflammatory cytokines and dysregulation of immune pathways are considered central to the disease process [1,4]. Genetic predisposition, particularly mutations in the gamma-secretase complex, has been implicated, but the genetic basis for sporadic HS cases is unclear [5,6].

One of the emerging areas of interest in the pathogenesis of inflammatory skin diseases like HS is pyroptosis, a form of programmed cell death distinct from apoptosis and necrosis [7]. Pyroptosis is characterized by its highly inflammatory nature, driven by the formation of membrane pores and the subsequent release of proinflammatory cytokines such as interleukin-1β (IL-1β) and interleukin-18 (IL-18) [7,8]. These cytokines are known to play a significant role in driving the inflammatory response in HS, contributing to the formation of abscesses and tunnels in the skin [1,4,6]. Gasdermins, a family of proteins, are central mediators of pyroptosis [9]. When activated, they form pores in the cell membrane, leading to the release of inflammatory mediators [10]. This process not only exacerbates local tissue inflammation but also recruits additional immune cells, perpetuating the cycle of inflammation [11]. The involvement of gasdermins in pyroptosis has been implicated in several chronic inflammatory diseases, including psoriasis, which shares pathogenetic similarities with HS [12]. In psoriasis, pyroptosis has been shown to contribute to activating proinflammatory pathways such as the IL-17 and IL-23 axes, which are also implicated in HS [12]. Given the involvement of pyroptosis in psoriasis, which shares many inflammatory pathways with HS, it is reasonable to hypothesize that targeting the gasdermin-mediated pyroptosis pathway could offer therapeutic potential in HS. Reducing pyroptotic cell death may help mitigate the excessive inflammation and tissue damage seen in HS patients, potentially improving clinical outcomes.

This study aimed to explore the potential involvement of gasdermins, namely gasdermin D (GSDMD) and gasdermin E (GSDME), in HS. We utilized enzyme-linked immunosorbent assay (ELISA) to assess their serum concentration and reverse transcription polymerase chain reaction in real time (RT-qPCR) to quantify the mRNA levels of *GSDMD* and *GSDME* in the skin samples. Understanding the association of gasdermins with HS could open new avenues for targeted therapies to modulate pyroptosis and control inflammation.

## 2. Materials and Methods

The research was conducted in accordance with the ethical principles of the Declaration of Helsinki and received approval from the Ethics Committee of Wroclaw Medical University (KB-750/2021, KB-779/2022, KB-250/2023, KB 265/2024). All patients provided written informed consent prior to their participation in the study.

### 2.1. Study Type and Design

This is a prospective cross-sectional study conducted to compare systemic protein concentrations (ELISA) and skin gene expression levels (RTqPCR) of GSDMD and GSMDE as well as to analyze their correlation with patient- and disease-related data.

**Figure 1:**
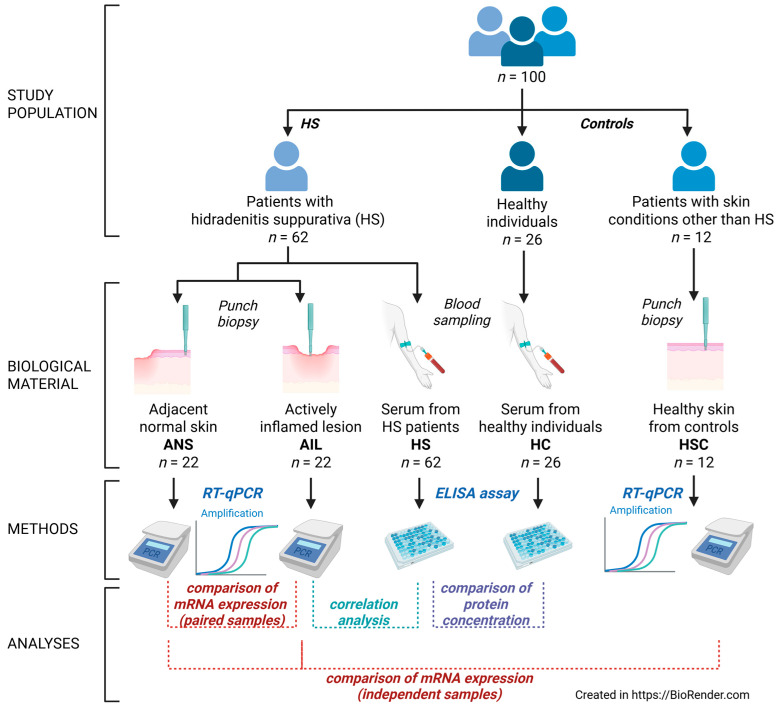
Diagram of study design.

One hundred individuals were recruited for the study, including sixty-two HS patients (study group) and thirty-eight subjects without HS, who formed two control groups. The first one consisted of 26 individuals and was used in protein analysis (further referred to as HC group). The second control group consisted of 12 individuals and was used in the skin expression analysis (HSC). Serum concentrations of gasdermins were determined in all HS patients while their gene expression was determined in a subgroup of 22. Skin fragments were collected from the actively inflamed lesions (AILs) as well as from patient-matched apparently healthy skin adjacent to the lesions (ANS). A diagram of study design is presented in Figure 1.

### 2.2. Study Population

#### 2.2.1. Study Group―Patients with Hidradenitis Suppurativa (HS)

HS patients (*n* = 62) were consecutively admitted between June 2022 and May 2024 to the Department of Dermatology, Venereology, and Allergology of Wroclaw Medical University for treatment. An experienced dermatologist, an expert in HS, performed comprehensive clinical evaluations. The demographic details such as age, sex, weight, height, body mass index (BMI), smoking habits, and HS-specific data (disease duration, age at onset, previous treatments, and surgical history) were collected. Routinely assessed laboratory data, such as whole blood cell count, concentrations of hemoglobin (Hb), fasting glucose (FG), insulin (FI), total cholesterol (tCHOL) and its LDL and HDL fractions, triacylglycerols (TG), uric acid (UA), urea, creatinine (CREA), total bilirubin (tBIL), C-reactive protein (CRP), ferritin, and iron and activities of aminotransferases AST and ALT, gamma-glutamyl transferase (GGT), and alkaline phosphatase (ALP), were retrieved from patients’ medical records.

The severity of HS was assessed using the Hurley staging system and the International Hidradenitis Suppurativa Severity Score System (IHS4) [13,14]. The Hurley system classifies HS into three stages: stage I (single or multiple abscesses without sinus tracts or scarring), stage II (recurrent abscesses with sinus tracts and scarring, but limited area involvement), and stage III (diffuse or multiple interconnected sinus tracts and abscesses with extensive scarring) [13]. The IHS4 provides a quantitative score based on the number of nodules, abscesses, and draining fistulas to classify the disease as mild, moderate, or severe [14].

Patients were categorized by BMI into three groups: normal weight (<25 kg/m^2^), overweight (25–29.9 kg/m^2^), and obese (≥30 kg/m^2^). Obesity was further graded as I (30–34.9 kg/m^2^), II (35–39.9 kg/m^2^), and III (≥40 kg/m^2^).

Hypercholesterolemia (hyperCHOL) and hypertriglyceridemia (hyperTG) were recognized if tCHOL ≥ 190 mg/dL and TG ≥ 100 mg/dL, respectively, and LDL cholesterol (LDL-C) was considered too high at 115 mg/dL while HDL cholesterol (HDL-C) was considered too low at <40 mg/dL for males and <45 mg/dL for females. Based on lipid profile, several indices used for assessing cardiometabolic health and cardiovascular risk were calculated, including: non-HDL cholesterol (non-HDL-C: tCHOL-HDL-C; cut-off: ≥130 mg/dL), Castelli’s risk index I (CRI-I: tCHOL/HDL-C; cut-off: >4.5 for females and >5 for males) and II (CRI-II: LDL-C/HDL-C; cut-off: >3 for females and >3.5 for males), atherogenic coefficient (AC: non-HDLchol/HDL-chol; cut-off: ≥3), lipoprotein combine index (LCI: tCHOL×TG×LDL-C/HDL-C; cut-off: ≥16), and atherogenic index of plasma (AIP: log10 (TG/HDL-C); cut-off: ≥0.11) [15].

Impaired fasting glucose (IFG) was recognized at ≥ 110 mg/dL. For hyperinsulinemia (HI), two thresholds of fasting insulin concentrations, namely ≥10 and 17 mU/mL, were applied. Insulin resistance (IR) was recognized using HOMA-IR and QUICKI indices with ≥2.5 and ≤0.33 scores used as thresholds. In addition, triglyceride–glucose index (TyG) was calculated as log 10 (TG×FG/2) and used as an IR indicator with a cut-off of ≥4.5. Hyperuricemia (hyperUA) was recognized if UA > 5 mg/dL in females and >6 mg/dL in males.

Anemia was recognized if Hb was ≤12 g/dL in females and ≤13 g/dL in males. Inflammation was recognized if CRP ≥ 10 mg/L. Based on whole blood cell counts, inflammation/immunity indices such as neutrophil-to-lymphocyte ratio (NLR; cut-off ≥ 2.8) and platelet-to-lymphocyte ratio (PLR) and systemic immune-inflammation index (SII) (PLT×NEU/LYM) were calculated as well.

#### 2.2.2. Control Groups

The control group for gasdermin protein determination consisted of 26 healthy volunteers (HC) whose serum samples were kindly provided by the Lower Silesian Blood Donation Center.

The control group for the analysis of gasdermin gene expression in the skin (HSC) consisted of 12 consecutive patients who underwent surgery for non-malignant skin conditions other than HS.

Control and study groups were well-matched with study groups in terms of median age and sex distribution (Table 1).

### 2.3. Biological Material

#### 2.3.1. Blood

A total of 9 mL of blood was drawn by venipuncture during routine sampling and at the time of biopsy. Blood was collected in serum separator tubes and left to clot for 30 min at room temperature. Samples were subsequently centrifuged for 15 min at 3000× *g*. Resulting sera were aliquoted and stored at −80 °C until analyses.

#### 2.3.2. Skin Biopsies

Punch biopsies of 5 mm were taken from actively inflamed lesions (AILs) and adjacent healthy-appearing skin (at least 2 cm from the lesions; ANS). The biopsies were performed after administration of local anesthesia using 2% lidocaine with adrenaline. They were immediately preserved in RNAlater (Sigma Aldrich, St. Luis, MO, USA) and stored at −80 °C until RNA extraction.

### 2.4. Analytical Methods

#### 2.4.1. Circulating GSDMD and GSDME

Serum concentrations of gasdermins were measured colorimetrically using enzyme-linked immunosorbent assays (ELISAs): Human Gasdermin D (#E6838Hu) from BT LAB (Korain Biotech, Jiaxing, China) and Human Gasdermin E (#AG-45B-00240KI01) from AdipoGen Life Sciences, Inc. (Füllinsdorf, Switzerland). The assays were conducted in accordance with manufacturer’s instructions. Color intensity, proportional to GSDMD and GSDME concentration, was measured at 450 nm using the EPOCH microplate reader (BioTEK^®^ Instruments, Inc., Winooski, VT, USA). GSDMD had a test range of 0.05–15 ng/mL with a sensitivity of 0.02 ng/mL, and GSDME had a test range of 0.06–4 ng/mL with a sensitivity of 0.05 ng/mL.

#### 2.4.2. Skin Expression of GSDMD and GSDME

RNA extraction and purification

Samples of up to 30 mg were thawed on ice and homogenized in Bead Elite Homogenizer (OMNI Internationale, Kennesaw, GA, USA) using ceramic spheres and a lysis buffer from a PureLink™ RNA Mini Kit (Invitrogen, Thermo Fisher Scientific, Waltham, MA, USA) with β-mercaptoethanol (Sigma Aldrich, St. Luis, MO, USA) at 1:10 (*v*/*v*). Phenol–chloroform extraction and the spin column method using a PureLink™ RNA Mini Kit (Invitrogen) were applied to isolate and purify RNA, respectively. In order to avoid contamination with genomic DNA, RNA isolates were subjected to on-column digestion with a PureLink™ DNase Set (Invitrogen). A NanoDrop 2000 spectrophotometer (Thermo-Fisher Scientific) and LabChip microfluidic technology, using the Experion platform and Experion RNA StdSens analysis kits (BioRad, Herkules, CA, USA), were utilized in the assessment of quantity, quality, and integrity of isolated RNA.

cDNA

An amount of 1000 ng per sample of isolated RNA was reversely transcribed on a C1000 thermocycler (BioRad) using an iScript™ cDNA Synthesis Kit (BioRad) according to the manufacturer’s instructions.

qPCR

Gene expression was quantified using a CFX96 Real-Time PCR system (BioRad) and 2×SsoFast EvaGreen^®^ Supermix (BioRad), applied in accordance with the provider’s instructions. The reaction mixture contained 10 µL of supermix and 1 µL each of 10 nM forward (F) and reverse (R) target-specific primers, 2 µL of 5-fold diluted (with water) cDNA template, and 6 µL of water. The following reaction conditions were set: 30 s of polymerase activation at 95 °C, 40 cycles comprising 5 s of denaturation at 95 °C and annealing and extension for 5 s at 61 °C. Melting curve analysis (60–95 °C, fluorescent readings every 0.5 °C) and agarose electrophoresis (SeaKem LE agarose from Lonza, Basel, Switzerland) with SYBR Green (Lonza) detection were utilized to verify the product’s specificity. Primers were synthesized by Genomed (Warsaw, Poland) based on sequences proposed by OriGene Technologies, Inc. (Rockville, MD, USA; www.origene.com, assessed on 17 October 2024). Primers span introns to further prevent accidental gDNA amplification.

Primer sequences for target genes (*GSDMD* and *GSDME*) and normalizers (YWHAZ, GUSB, and GAPDH) are presented in Table 2.

Arithmetic means of Cq values from technical replicates were calculated and their geometric mean for a given sample set was obtained. This geometric mean was subtracted from Cqs of the individual samples. Resulting ΔCq values were then linearized by 2^^ΔCq^ conversion and normalized to the geometric mean of three reference genes. The values calculated are further referred to as a “normalized relative quantity” (NRQ) [16] and used in statistical analysis.

### 2.5. Statistical Analysis

Data distribution was tested using the Shapiro–Wilk test and homogeneity of variances was analyzed using either an *F*-test for two-group comparisons or Levene’s test for homogeneity of error variances for multi-group comparisons. To normalize data distribution, if required, data were log-transformed. Comparisons of means between two groups were conducted with a *t*-test for independent samples and presented as arithmetic (non-transformed data) or geometric means (log-transformed data) with a 95% confidence interval (CI). In the case of non-normally distributed data or inequality of variances, they were analyzed using a Mann–Whitney *U* test and presented as medians with 95% CI. For multi-group comparisons, a Kruskal–Wallis *H* test with a Conover post hoc test was applied. For pair-wise analyses, a *t*-test for paired samples or non-parametric Wilcoxon test was used. Depending on data size, character, and distribution, correlation analyses were conducted using Pearson’s product moment correlation and Spearman or Kendall rank correlation tests. Their results were presented as correlation coefficients *r*, *ρ*, or *τ*, respectively.

All analyses were conducted using MedCalc^®^ Statistical Software version 23.0.2 (MedCalc Software Ltd., Ostend, Belgium; https://www.medcalc.org; 2024) licensed to Prof. Malgorzata Krzystek-Korpacka. Statistical significance was set at probability (*p*) < 0.05 and calculated *p* values were two-tailed.

## 3. Results

### 3.1. Characteristics of HS Patients

The study group comprised 42% females and the median age of HS patients was 37 years, with no significant difference between males and females. One-third of patients were classified as having severe disease based on the Hurley score and over 70% based on the IHS4 score. On average, HS duration was 6 years and over half of patients had a history of smoking, were insulin-resistant, and had elevated BMI as well as concentrations of non-HDL cholesterol and triacyclglycerols. None of these parameters differed between sexes; however, substantially and significantly more males were at elevated cardiovascular risk as indicated by AIP and AC indices (Table 3). Detailed group characteristics indicated also slightly elevated CREA, UA, ALT, GGT, and ALP as well as lower HDL-C in male HS patients without any significant sex-related differences in other parameters (Table 3).

### 3.2. Gasdermins in HS Patients and Healthy Controls

**Figure 2:**
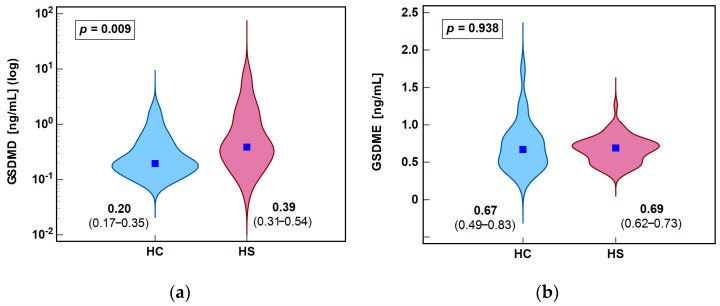
Circulating gasdermins in patients with hidradenitis suppurativa (HS) and healthy controls (HC): (**a**) GSDMD; (**b**) GSDME. Data were analyzed using Mann–Whitney *U* test. Test results are presented as medians with 95% CI and probability *p*. Data distribution is illustrated by violin plots with dark blue solid squares marking median values. CI, confidence interval.

Median GSDMD concentration was significantly higher in the HS than HC group. In contrast, GSDME concentrations showed no significant difference (Figure 2).

### 3.3. Impact of Patients’ and the Disease Characteristics on Circulating Gasdermins

Neither GSDMD nor GSDME differed significantly between HS and HC groups by sex, smoking habit or HS severity in terms of Hurley score or IHS4; although GSDMD tended to be lower in patients with higher IHS4 scores. Its concentrations were higher in female patients with elevated lipid-based markers of cardiovascular risk, significantly so in the case of AC, AIP, and LCI indices (Table 4).

There were also sex-specific differences in GSDME concentrations, depending on the absence or presence of certain metabolic disorders. Circulating GSDME was significantly lower in females with IR indicated by the TyG index, hypercholesterolemia, elevated non-HDL cholesterol, hypertriglyceridemia, or elevated cardiovascular risk indicated by LCI. It also tended to be decreased in overweight/obese females, with elevated LDL cholesterol or cardiovascular risk indicated by AC. Significantly lower GSDME also accompanied anemia independently from sex (Table 4). There were no associations with other metabolic abnormalities in females and no associations at all among male patients (Supplementary Materials: Table S1), except for lower GSDME concentrations in males with low-grade inflammation by CRP (Table 4).

Neither GSDMD nor GSDME correlated with patients’ age, IHS4 score, or the disease duration (Table 5).

Correlation analysis revealed a significant negative relationship between circulating GSDMD and RBC and BMI as well as a tendency—in female HS patients—towards a negative relation with ALP activity and a positive one with TG. In turn, GSDMD in females positively correlated with serum concentrations of ferritin and iron (Table 5).

Circulating GSDME, in turn, was positively correlated with creatinine and with Hb concentration, significantly so in males, in whom it also tended to correlate with iron. In addition, it correlated negatively with glucose in females while this relationship in males tended to be positive (Table 5) and gained statistical significance when a more sensitive Kendall rank test was applied (*τ* = 0.38, *p* = 0.037). Likewise, GSDME tended to be inversely related to AST activity in females, significantly so in the Kendall test (*τ* = −0.35, *p* = 0.035). In females, serum GSDME concentrations correlated negatively with an IR index—TyG—cardiovascular risk index LCI, and concentrations of TG, CHOL, and LDL-CHOL and tended to be inversely related with non-HDL cholesterol and cardiovascular risk by AIP (Table 5).

No correlation between circulating gasdermins and inflammatory/immune markers or any other biochemical parameters could be observed (Supplementary Materials: Table S2).

Serum GSDMD and GSDME concentrations in healthy individuals were not affected by their age (*τ* = 0.21, *p* = 0.132 and *τ* = 0.09, *p* = 0.549) or sex (*p* = 0.940 and *p* = 0.227).

### 3.4. mRNA Expression of GSDMD and GSDME in the Skin

**Figure 3:**
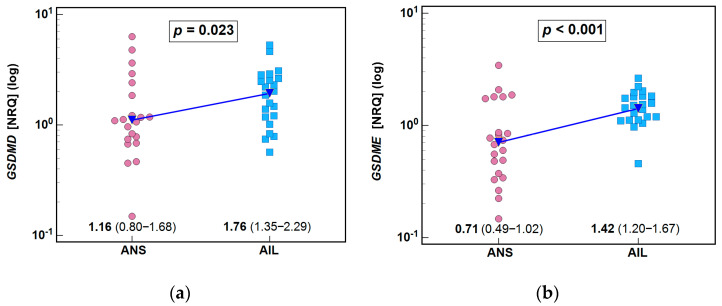
Expression of gasdermins in the skin of HS patients: (**a**) *GSDMD*; (**b**) *GSDME*. Data were analyzed with *t*-test for paired samples and are presented as means with 95% CI. AIL, actively inflamed lesions; ANS, patient-matched adjacent healthy-appearing skin; CI, confidence interval; NRQ, normalized relative quantities.

A pairwise comparison of *GSDMD* and *GSDME* mRNA levels in actively inflamed lesions (AILs) and adjacent healthy-appearing skin (ANS) showed their significant upregulation in AILs, by 1.5-fold for *GSDMD* and by 2.0-fold for *GSDME* (Figure 3).

**Figure 4:**
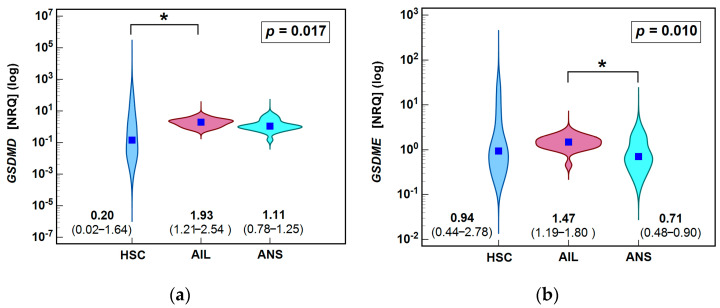
Expression of gasdermins in the skin of HS patients and control individuals: (**a**) *GSDMD*; (**b**) *GSDME*. Data presented in the form of violin plots. Individual median values with 95% confidence interval are given below the plots and additionally marked by dark blue squares. Data were analyzed using Kruskal–Wallis H test with post hoc Conover test. Significant (*p* < 0.05) between-group differences are indicated by (*). AIL, actively inflamed lesions; ANS, patient-matched adjacent healthy-appearing skin; NRQ, normalized relative quantities; HSC, healthy skin from controls; *p*, probability.

Gasdermins’ expression in HS patients, both in ANS and AILs, was further compared to their expression in healthy skin fragments from control individuals (HSC). The mRNA expression of *GSDMD* was significantly higher in AILs as compared to HSC while *GSDME* expression was comparable between HSC and AILs and between HSC and ANS (Figure 4).

### 3.5. Association of Patients’ and Disease Characteristics with Skin Expression of GSDMD and GSDME

#### 3.5.1. GSDMD

Local *GSDMD* expression in the skin was not affected by patients’ sex and smoking habits or the disease severity and duration, whether it was analyzed in lesions (AILs) or nearby normal-appearing (ANS) skin fragments or as an AIL-to-ANS ratio (fold change in expression) (Table 6). Skin *GSDMD* expression was not associated with the presence of inflammatory/immune responses or metabolic abnormalities (Supplementary Materials: Table S3), except for its lower level in AILs from HS patients with hyperuricemia (Table 6).

Skin expression of *GSDMD* mRNA did not correlate with age, IHS4, or HS duration but in AILs it tended to be inversely related to serum uric acid and directly to GGT activity. In turn, *GSDMD* in ANS tended to positively correlate with serum bilirubin, contributing to a negative association between bilirubin and fold change in *GSDMD* expression but also lacking statistical significance (Table 7).

No correlations with other markers and indices could be found (Supplementary Materials: Table S4).

*GSDMD* expression in normal skin fragments from individuals without HS (HSC) was negatively affected by their age (*τ* = −0.55, *p* = 0.010) but not sex (*p* = 0.745).

#### 3.5.2. GSDME

Local *GSDME* expression in the skin was not affected by patients’ sex and smoking habits or the disease severity and duration, whether it was analyzed in AILs or ANS or as a fold change in expression. Of evaluated metabolic abnormalities, *GSDME* expression in AILs tended to be lower in patients with HDL cholesterol below the norm. *GSDME* expression in ANS was 2.6 times higher in patients with elevated CRI2 and AIP scores, significantly so in the case of AIP. Consistently, fold change in gene expression between AILs and ANS for high-risk patients was 2.3 times lower. In addition, fold change in GSDME expression between lesions and normal skin was, significantly, 2.4 times lower in patients with hypertriglyceridemia and tended to be 2.5 times lower in the case of insulin resistance (Table 8).

Skin *GSDME* expression was not associated with the presence of inflammatory/immune responses or any other metabolic abnormalities (Supplementary Materials: Table S5).

However, its expression in AILs tended to correlate negatively with LCI while the fold change in *GSDME* expression between tissues correlated with HOMA-IR and CRI-2 indices. In turn, both expression in ANS (positively) and fold change (negatively) correlated significantly with ferritin concentration and patient’s age. Moreover, GSDME expression in ANS tended to decrease with IHS4 score but increase with the disease duration. Fold change, in turn, decreased along with increasing IgA concentrations (Table 9).

No correlations with other markers and indices could be found (Supplementary Materials: Table S6).

*GSDME* expression in normal skin fragments from individuals without HS (HSC) was not affected by their age (*τ* = −0.34, *p* = 0.109) or sex (*p* = 0.635).

### 3.6. Interplay Between Systemic and Local Gasdermins

There was no correlation between circulating GSDMD and GSDME in healthy controls (*r* = 0.26, *p* = 0.197) and HS patients (*r* = −0.12, *p* = 0.342) as well as between their local expressions in AILs (*r* = 0.33, *p* = 0.135). However, *GSDMD* and *GSDME* expression tended to be positively correlated in patient-matched ANS (*r* = 0.38, *p* = 0.081) and was directly and significantly correlated in normal skin from individuals without HS (*r* = 0.58, *p* = 0.049).

Circulating GSDME correlated positively and significantly with its local expression in AILs (*r* = 0.50, *p* = 0.020) but not in ANS (*r* = 0.07, *p* = 0.765). There was also no correlation with the fold change in *GSDME* expression (*r* = 0.18, *p* = 0.436).

Circulating GSDMD did not correlate with its local expression in AILs (*r* = −0.04, *p* = 0.850) and ANS (*r* = 0.22, *p* = 0.348) or with the fold change in *GSDMD* expression (*r* = −0.25, *p* = 0.266).

## 4. Discussion

HS is a chronic inflammatory disease characterized by recurrent nodules, abscesses, and sinus tract formation in intertriginous areas [1]. Despite the advances in understanding the inflammatory and immune pathways involved in HS, the specific molecular mechanisms contributing to the pathology remain incompletely understood [17,18,19].

Our study provides important insights into the role of pyroptosis in HS by evaluating gene expression for *GSDMD* and *GSDME* in both lesional and non-lesional skin, as well as determining serum concentrations of both gasdermins and placing the findings in a broad clinical context. We observed significantly elevated levels of GSDMD in the serum of HS patients compared to healthy controls, confirming the involvement of pyroptosis in driving the inflammatory response. Additionally, tissue analysis revealed that both *GSDMD* and *GSDME* mRNA expression were significantly upregulated in lesional skin compared to patient-matched non-lesional skin, suggesting localized activation of pyroptosis in areas of active inflammation. The increased *GSDMD* mRNA in HS lesions, particularly in the absence of a strong correlation with clinical severity (Hurley stage or IHS4), implies that pyroptosis may be more involved in disease initiation and maintenance rather than progression. Lack of association between GSDMD, both systemic and local, and markers of inflammation and immunity demonstrated in this study seem to corroborate the notion. While *GSDME* mRNA expression was also increased in lesional skin, it did not show significant differences in serum levels between HS patients and controls, indicating that *GSDME* may play a more limited or localized role in the inflammatory cascade and/or be involved in activities unrelated to pyroptosis. Significantly, the lesional expression of *GSDMD*, but not *GSDME*, was also upregulated compared to normal skin from individuals not suffering from HS. These findings underscore the importance of *GSDMD* as a more significant mediator of inflammation and tissue destruction in HS.

Pyroptosis amplifies the inflammatory environment by releasing proinflammatory cytokines, particularly IL-1β and IL-18, from ruptured cells [12,20]. These cytokines further recruit immune cells to the site of inflammation, exacerbating the cycle of immune activation and tissue damage [1,21,22]. Indeed, transcriptomic analysis of HS lesional and perilesional skin—published by our team in 2024 [6]—identified profound enrichment of inflammatory-related genes in HS lesions compared to both perilesional and control skin. RNA sequencing data showed that genes encoding proinflammatory cytokines, chemokines, and mediators of immune cell chemotaxis were highly upregulated in HS lesions [6]. Specifically, genes associated with granulocyte migration, neutrophil chemotaxis (e.g., IL-1β, CXCL1-3, IL-17A/F, S100A7/8/9), and bacterial response were elevated, further supporting the contribution of pyroptosis to HS pathogenesis [6]. According to the available studies, the upregulation of *GSDMD* in lesional skin correlates with increased infiltration of immune cells, abscess formation, and the development of tunnels, all of which are hallmark features of HS [1,21]. This suggests that GSDMD-induced pyroptosis may drive inflammation and serve as a key initiator of the tissue damage that leads to abscess and tunnel formation. The inability to downregulate pyroptosis in HS lesions may contribute to the chronicity of the disease, as damaged tissue fails to resolve inflammation and instead forms fibrosis and scarring [1]. We showed that, unlike locally, systemic immune cell counts correlate neither with circulating GSDMD nor its skin expression. Instead, serum GSDMD was inversely related to erythrocyte count, which is consistent with observation on the gasdermin knockout preventing anemia development in animal models of some inflammatory diseases [23,24]. The possible mechanism involves GSDMD-mediated upregulation of IL-1β resulting in diminished erythropoiesis and an increase in iron labile pool and ferritin expression [25]. Consistently, circulating GSDMD positively correlated with concentrations of both ferritin and iron in our female HS patients. Local *GSDMD* expression in lesions was inversely related to uric acid, which seems to be counterintuitive because of transcriptional regulation of this gasdermin by the NFκB pathway, activated by uric acid—a damage-associated molecular pattern (DAMP) [26]. However, the observed phenomenon might represent a feedback inhibition mediated by cytokines and/or result from uric-acid-mediated epigenetic regulation of genes encoding immune and inflammatory mediators [27]. Indeed, hypermethylation of the GSDMD promoter is known to suppress gasdermin expression [26].

HS shares many inflammatory pathways with psoriasis [28]. Both diseases exhibit elevated levels of IL-1β and IL-18, which are key mediators of pyroptosis [29]. However, there are essential differences in how pyroptosis manifests between these diseases. In psoriasis, pyroptosis occurs primarily in keratinocytes, contributing to the hyperproliferation and plaque formation typical of the disease [12]. In HS, pyroptosis is more localized to the hair follicle and surrounding skin, where it drives the formation of abscesses, tunnels, and extensive scarring [12]. The absence of a strong correlation between GSDMD levels and clinical severity in HS contrasts with findings in psoriasis, where pyroptosis directly contributes to disease progression [30]. This suggests that, while pyroptosis is important in the pathogenesis of both conditions, the details of GSDMD involvement may differ. Corroborating the notion, therapies targeting pyroptosis, such as IL-1β inhibitors, have shown moderate success in psoriasis, but the same treatments have had limited efficacy in HS, likely due to differences in the tissue-specific manifestation of inflammation [12].

Elevated GSDMD levels in HS suggest that targeting the pyroptotic pathway could represent a novel therapeutic strategy. GSDMD plays a central role in forming membrane pores that allow the release of proinflammatory cytokines [31]. Inhibitors of GSDMD, such as disulfiram, which has shown promise in other inflammatory diseases, could be repurposed for the treatment of HS [32]. By inhibiting GSDMD, it may be possible to reduce the release of IL-1β and IL-18, thereby dampening the inflammatory response and preventing the progression of lesions into chronic, scarring abscesses and tunnels [32]. In addition to direct inhibition of GSDMD, upstream mediators of pyroptosis, such as the NLRP3 inflammasome and caspase-1, present attractive therapeutic targets. Inhibitors of the NLRP3 inflammasome, like MCC950, have demonstrated efficacy in preclinical models of other chronic inflammatory diseases and could similarly reduce pyroptotic cell death in HS [33]. Caspase-1 inhibitors, which prevent the cleavage of GSDMD and the activation of IL-1β, also hold potential as therapeutic agents [34]. These therapies could be particularly beneficial for patients with severe or recalcitrant disease, where conventional treatments, such as TNF-α inhibitors, are less effective. Furthermore, biologic therapies targeting IL-1β, such as anakinra and canakinumab, have been explored in HS. Anakinra, an IL-1 receptor antagonist, has shown variable efficacy, potentially due to differences in the underlying inflammatory pathways driving disease in different patient subgroups [35,36]. Canakinumab, a monoclonal antibody against IL-1β, has been used in small case series with inconsistent outcomes [37,38]. Still, these treatments may benefit from combination with GSDMD or caspase-1 inhibitors to more comprehensively block pyroptosis and reduce inflammation.

GSDME shares a pore-forming ability with GSDMD but the mechanisms of pyroptosis conducted by the two gasdermins differ, e.g., GSDME-mediated pyroptosis is inflammasome-independent, requires caspase-3 or granzyme B to activate GSDME, and may not lead to target cell lysis [39]. Although there is a cross-talk between pyroptotic pathways mediated by both gasdermins [39], we found no correlation between circulating GSDMD and GSDME both in HS patients and healthy controls. There was also no correlation between their expression in HS lesions. However, there was a tendency towards a positive correlation in patient-matched normal skin as well as a significant positive correlation in normal skin from individuals without HS. This observation further supports the notion of distinct roles played by these gasdermins in HS. It is also evidenced by distinct patterns of GSDMD and GSDME association with metabolic disorders. Their concentrations were particularly affected by cardiovascular risk, which, in GSDMD’s case, was expressed in terms of lipid-derived indices such as AC, AIP, LCI, and CRI-1. In GSDME’s case, its concentration correlated with cardiovascular risk by LCI and with dyslipidemia manifested by hypercholesterolemia and hypertriglyceridemia. Intriguingly, gasdermins’ association with these metabolic disorders was present solely in female HS patients, though they were at significantly lower cardiovascular risk on average than males. However, while GSDMD was expectedly elevated, GSDME was significantly lower. Circulating GSDME was also consistently lower in HS females with deregulated carbohydrate metabolism, namely with coexisting insulin resistance, in whom the gasdermin was inversely correlated with glucose and TyG—an insulin resistance index. An increase in GSDMD is to be expected considering its contribution to low-grade inflammation underlying cardiometabolic disorders as well as an established ability to dysregulate in lipid and carbohydrate pathways to trigger cell death by pyroptosis [40]. Likewise, GSDME partakes in the pathogenesis of cardiometabolic diseases and its expression is triggered by a rise in concentrations of small oxidized LDL particles, among others [41]. In this light, lower serum concentrations of GSDME in HS patients burdened with metabolic deregulations, mirrored by reduced fold change in local *GSDME* expression, may come as a surprise. Unfortunately, there seems to be a gap in the clinical data regarding GSDME in metabolic disorders. Nonetheless, a negative correlation between serum GSDME and BMI, glucose, and triacylglycerols has also recently been reported in psoriasis [42]. Moreover, psoriatic patients had only slightly elevated circulating GSDME compared to healthy controls [42]. Furthermore, GSDME ablation hampered lipolysis and increased susceptibility of tested animals to obesity and insulin resistance, as recently reported by Wei et al. [43], implying a beneficial role of GSDME in this setting. The possible link between metabolic abnormalities and GSDME warrants closer examination. Assuming its negative impact, it may in fact counteract and mask potential GSDME elevation in serum in response to HS, which would be consistent with an upregulation of its local expression. Resolving the relationship between metabolic disorders and GSDME as well as the role of sex is of utmost clinical importance in view of their prevalence in HS, significantly higher than in the general population [44,45,46], and emerging treatment strategies involving targeting gasdermins.

## 5. Limitations

While this study provides valuable insights into the involvement of key gasdermins in HS, it is important to acknowledge several limitations.

First, the sample size, while relatively modest, was sufficient to detect statistically significant differences in gasdermin levels between HS patients and healthy controls. Although larger studies would further strengthen the generalizability of these results, the consistency of our findings with previously published research supports the validity of our conclusions. The inclusion of both normal skin fragments from healthy controls and patient-matched lesional and non-lesional samples also provides robust comparative data that mitigates concerns about sample size.

Second, our study primarily focused on GSDMD and GSDME, key mediators of pyroptosis. While other cell death pathways, such as necroptosis and apoptosis, may also contribute to HS pathogenesis, the specific focus on pyroptosis allowed for a detailed investigation of this underexplored mechanism. Future studies may indeed broaden the scope to include other pathways. Still, the findings from this study provide a solid foundation for understanding the relevance of pyroptosis in HS, which is a promising target for therapeutic intervention.

Thirdly, while limiting our ability to track temporal changes in gasdermin expression, the study’s cross-sectional design still provides valuable insights into the association between pyroptosis and inflammation in both early and chronic HS lesions. Longitudinal studies could build upon this work to determine the exact timing of pyroptosis activation. Still, the consistent upregulation of gasdermins in lesional tissue strongly suggests their involvement throughout the disease process.

Ultimately, a key limitation of our study is the lack of direct assessment of GSDMD and GSDME protein expression in skin tissues, such as through Western blot or immunohistochemistry. While protein-level analyses could further confirm gasdermin involvement, previous research across various inflammatory diseases, including psoriasis, atopic dermatitis, or atherosclerosis [41,47,48], consistently shows a strong correlation between increased gasdermin mRNA expression and corresponding protein levels. Therefore, our findings—significantly elevated GSDMD serum levels in HS patients and increased tissue mRNA expression of both GSDMD and GSDME in lesional skin—remain robust and align well with existing literature. Nevertheless, future studies incorporating protein-level analyses are necessary to clarify further the precise role and activation status of gasdermins in the pathogenesis of HS.

## 6. Conclusions

In conclusion, our study demonstrates that pyroptosis, mediated by GSDMD and GSDME, plays a significant role in the pathogenesis of HS. Enhanced *GSDMD* mRNA expression in lesional skin suggests that pyroptosis is a key driver of the excessive inflammation and tissue damage characteristic of HS. Targeting pyroptosis through inhibitors of GSDMD, caspase-1, or the NLRP3 inflammasome represents a promising therapeutic approach for reducing inflammation and improving clinical outcomes in HS patients. GSDME, in turn, seems to be more involved in metabolic comorbidities and plays a positive role, which ought to be taken into consideration while devising gasdermin-targeted anti-HS therapies. Further research is needed to fully elucidate the mechanisms underlying pyroptosis in HS and to develop effective therapies for this challenging condition.

## Figures and Tables

**Table 1:** Comparison of test and control groups by sex and age in cohorts for analyses of protein and gene expression.

Factor	Protein Analysis			Gene Expression Analysis		
	HC	HS	*P*	Controls	HS	*P*
Sex [F/M], *n* Females	14/12 54%	26/36 42%	0.353 ^1^	4/8 58%	8/14 36%	1.0 ^1^
Age [yrs.] median (95% CI)	42 (31–49)	37 (32–44)	0.253 ^2^	57 (20–73)	43 (27–49)	0.341 ^2^

^1^, Fisher’s exact test (2 × 2); ^2^, Mann–Whitney *U* test; HC, healthy controls; HS, patients with hidradenitis suppurativa; *P*, probability; *n*, number of cases; F/M; female-to-male ratio; yrs., years; CI, confidence interval.

**Table 2:** Characteristics of primers.

Gene	Encoded Protein	Primer Sequence (5′→3′)	Amplicon Size [bp]
*GSDMD*	Gasdermin D	F: atgaggtgcctccacaacttcc R: ccagttccttggagatggtctc	109
*GSDME*	Gasdermin E	F: gatctctgagcacatgcaggtc R: gttggagtccttggtgacattcc	112
*YWHAZ*	Tyrosine 3-monooxygenase/tryptophan 5-monooxygenase activation protein ζ	F: accgttacttggctgaggttgc R: cccagtctgataggatgtgttgg	130
*GUSB*	β-Glucuronidase	F: ctgtcaccaagagccagttcct R: ggttgaagtccttcaccagcag	126
*GAPDH*	Glyceraldehyde-3-phosphate dehydrogenase	F: gtctcctctgacttcaacagcg R: accaccctgttgctgtagccaa	131

bp, base pairs; F, forward primer; R, reverse primer.

**Table 3:** Characteristics of the study group as a whole and by sex.

Factor	Patients with HS			*p* Value ^1^
	All	Females	Males	
*N*	62	26 (42%)	36 (58%)	-
Age [yrs.]	37 (32–44)	35 (31–45)	39 (29.5–46.5)	0.849 ^2^
BMI [kg/m^2^]	29 (28–34)	30.5 (24–35)	29 (26–35)	0.678 ^2^
BMI [%] overweight/obese	27.9/48.8	15/55	39.1/43.5	0.197 ^3^
BMI abnormal [%]	76.7	70	82.6	0.473 ^4^
Severe HS by Hurley [%]	33.3	36.4	30.8	0.764 ^4^
IHS4	16 (14–22.5)	15 (10–24)	18.5 (15–24)	0.474 ^2^
Severe HS by IHS4 [%]	70.8	59.1	80.8	0.122 ^4^
Disease duration [yrs.]	6 (5–9)	7 (5–11.5)	6 (3–10)	0.496 ^2^
Smoking habit [%] yes/in the past	52.5/10	44.4/11.1	59.1/9.1	0.649 ^3^
Anemia [%]	13.6	10	16.7	0.673 ^4^
Glucose [mg/dL]	88.5 (81–94)	92 (81.5–108.5)	86 (77–93)	0.273 ^2^
IFG [%]	25	40	11.8	0.106 ^4^
Insulin [mU/mL]	14.1 (11.9–18.1)	16 (12.1–52.7)	13.5 (9.65–47)	0.341 ^2^
HI: >10 mU/mL [%]	75	90/40	64.3	0.341 ^3^
HOMA-IR	3.25 (2.2–4.62)	3.35 (2.94–31.69)	2.55 (2.08–11.1)	0.260 ^2^
IR by HOMA [%]	68.2	87.5	57.1	0.193 ^4^
TyG	4.59 (4.48–4.7)	4.53 (4.37–4.69)	4.65 (4.49–4.82)	0.255 ^5^
IR by TyG ≥ 4.5	51.6	40	62.5	0.289 ^4^
tCHOL [mg/dL]	184 (172–196)	180 (165–196)	188 (167–208)	0.557 ^5^
HyperCHOL [%]	35.3	33.3	37.5	1.0 ^4^
HDL-CHOL [mg/dL]	48.5 (43.6–53.3)	54.9 (48–61.9)	41.6 (36.1–47)	0.003 ^5^
↓ HDL-CHOL [%]	42.4	29.4	56.2	0.166 ^4^
LDL-CHOL [mg/dL]	110 (98–122)	105 (92–118)	117 (93–141)	0.348 ^5^
↑ LDL-CHOL [%]	43.3	35.3	53.8	0.460 ^4^
Non-HDL-CHOL [mg/dL]	137 (124–150)	129 (112–145)	146 (125–167)	0.176 ^5^
↑ Non-HDL-CHOL [%]	55.9	44.4	68.7	0.185 ^4^
TG [mg/dL]	101 (86–120)	92.5 (69–115)	116 (85–157)	0.143 ^2^
HyperTG [%]	52.9	44.4	62.5	0.327 ^4^
↑ CV risk by AIP [%]	30.3	11.8	50	0.025 ^4^
↑ CV risk by AC [%]	48.5	23.5	75	0.005 ^4^
↑ CV risk by CRI-I [%]	30.3	17.6	43.7	0.141 ^4^
↑ CV risk by CRI-II [%]	21.2	11.8	31.2	0.225 ^4^
↑ CV risk by LCI [%]	39.4	23.5	56.2	0.080 ^4^
CREA [mg/dL]	0.76 (0.73–0.79)	0.73 (0.68–0.77)	0.78 (0.75–0.82)	0.041 ^5^
Urea [mg/dL]	23.6 (21.3–25.9)	24.5 (21.3–27.7)	22.7 (19.2–26.2)	0.428 ^5^
UA [mg/dL]	5.46 (4.98–5.94)	4.87 (4.21–5.54)	6.05 (5.42–6.68)	0.010 ^5^
HyperUA [%]	40	33.3	46.7	0.710 ^5^
WBC [×10^3^/μL]	8.16 (7.34–9.07)	8.19 (6.81–9.85)	8.13 (7.13–9.27)	0.731 ^5^
Leukocytosis [%]	25	20	29.2	0.728 ^4^
NEU [×10^3^/μL]	5.19 (4.83–5.8)	5.31 (4.38–5.8)	5.0 (4.69–6.12)	0.906 ^2^
Neutrophilia [%]	13.6	10	16.7	0.673 ^4^
LYM [×10^3^/μL]	2.09 (1.84–2.36)	2.26 (1.74–2.79)	2.08 (1.8–2.32)	0.582 ^2^
PLT [×10^3^/μL]	282 (251–303)	277 (239–345)	287 (246–306)	0.841 ^2^
NLR	2.3 (2.0–2.8)	2.1 (1.7–2.9	2.3 (1.7–3.0)	0.723 ^2^
↑ NLR [%]	34.9	31.6	37.5	0.755 ^4^
PLR	133 (119–149)	131 (111–155)	134 (114–158)	0.837 ^5^
SII	681 (567–819)	685 (503–933)	678 (531–866)	0.960 ^5^
CRP [mg/L]	6.2 (4.5–9.2)	6.6 (2.7–10.5)	5.6 (4.4–10.5)	0.764 ^2^
Inflammation by CRP [%]	38.9	50	27.8	0.196 ^4^
tBIL [mg/dL]	0.46 (0.35–0.5)	0.4 (0.3–0.5)	0.5 (0.37–0.6)	0.177 ^2^
ALT [U/L]	20 (18–25.5)	17 (10.8–20.2)	25 (19–31.2)	0.014 ^2^
AST [U/L]	18 (15.5–19)	17 (13.8–19.2)	19 (16.6–23.2)	0.364 ^2^
GGT [U/L]	24 (22–28.9)	22 (16.8–28.2)	27.5 (23–52.4)	0.018 ^2^
ALP [U/L]	69.1 (61.8–77.2)	61.7 (53.2–71.6)	78.7 (67.3–92)	0.024 ^5^

^1^, for comparison of groups by sex; ^2^, data presented as medians (95% CI) and analyzed with Mann–Whitney U test; ^3^, Chi-squared test; ^4^, Fisher exact test; ^5^, data presented as means (95% CI) and analyzed with *t*-test for independent samples. AC, atherogenic coefficient; AIP, atherogenic index of plasma; ALP, alkaline phosphatase; ALT, alanine transaminase; AST, asparagine transaminase; BMI, body mass index; CHOL, cholesterol; CI, confidence interval; CREA, creatinine; CRI, Castelli’s risk index; CRP, C-reactive protein; CV risk, cardiovascular risk; GGT, gamma-glutamyl transferase; HI, hyperinsulinemia; HOMA-IR, homeostasis model assessment of insulin resistance; HS, hidradenitis suppurativa; IFG, impaired fasting glucose; IHS4, international hidradenitis suppurativa severity score system; IR, insulin resistance; LCI, lipoprotein combine index; LYM, lymphocyte count; N, number of cases; NEU, neutrophil count; NLR, neutrophil-to-lymphocyte ratio; *p*, probability; PLR, platelet-to-lymphocyte ratio; PLT, platelet count; SII, systemic immune-inflammation index; tBIL, total bilirubin; tCHOL, total cholesterol; TG, triacylglycerols; TyG, triglyceride-to-glucose index; UA, uric acid; WBC, leukocyte count; yrs., years; ↑, elevated; ↓, decreased.

**Table 4:** Impact of selected patients’ and disease characteristics on circulating gasdermins.

Factor	Categories	GSDMD		GSDME	
		Median	*p* Value	Median	*p* Value
Sex	females	0.34 (0.25–0.54)	0.364	0.62 (0.5–0.73)	0.221
	males	0.31 (0.20–0.45)		0.72 (0.63–0.77)	
Hurley score	1/2	0.42 (0.32–0.67)	0.793	0.71 (0.61–0.77)	0.205
	3	0.34 (0.25–1.32)		0.62 (0.5–0.72)	
HS severity by IHS4	moderate	0.58 (0.32–1.56)	0.063	0.56 (0.46–0.79)	0.352
	severe	0.33 (0.26–0.54)		0.69 (0.62–0.76)	
Smoking habit	no	0.54 (0.31–1.49)	0.404	0.72 (0.47–0.76)	0.716
	yes	0.34 (0.27–0.59)		0.70 (0.59–0.77)	
	in the past	0.38 (0.19–0.80)		0.73 (0.57–0.84)	
BMI	normal	0.47 (0.35–1.13)	0.438	0.76 (0.64–0.82)	0.093
	elevated	0.34 (0.29–0.74)		0.65 (0.54–0.74)	
Anemia	no	0.39 (0.32–0.62)	0.811	0.72 (0.65–0.76)	0.005
	yes	0.38 (0.17–6.19)		0.48 (0.37–0.61)	
IR (by TyG) F	no	0.36 (0.19–1.31)	0.594	0.76 (0.61–0.9)	0.007
	yes	0.98 (0.09–1.89)		0.48 (0.43–0.63)	
HyperCHOL F	no	0.43 (0.2–1.08)	0.281	0.71 (0.6–0.79)	0.031
	yes	0.99 (0.3–1.92)		0.48 (0.43–0.85)	
↑ LDL-CHOL F	no	0.36 (0.17–1.34)	0.546	0.72 (0.58–0.8)	0.063
	yes	0.74 (0.3–1.88)		0.52 (0.43–0.85)	
↑ non-HDL-CHOL F	no	0.34 (0.16–1.26)	0.230	0.73 (0.62–0.81)	0.029
	yes	0.76 (0.31–1.47)		0.54 (0.46–0.71)	
HyperTG F	no	0.41 (0.23–1.26)	0.450	0.73 (0.59–0.81)	0.050
	yes	0.76 (0.27–1.47)		0.54 (0.46–0.72)	
↑ CV risk by CRI-1 F	no	0.34 (0.28–1.13)	0.068	0.71 (0.48–0.79)	0.283
	yes	1.34 (0.8–1.89)		0.58 (0.52–0.59)	
↑ CV risk by AC F	no	0.32 (0.24–1.0)	0.047	0.72 (0.54–0.81)	0.089
	yes	1.23 (0.87–1.7)		0.54 (0.48–0.59)	
↑ CV risk by AIP F	no	0.36 (0.29–1.05)	0.044	0.7 (0.51–0.78)	0.296
	yes	1.7 (1.34–2.06)		0.54 (0.5–0.58)	
↑ CV risk by LCI F	no	0.32 (0.23–0.84)	0.036	0.72 (0.50–0.81)	0.023
	yes	1.23 (0.98–1.66)		0.48 (0.46–0.54)	
Inflammation by CRP M	no	0.32 (0.13–0.76)	0.218	0.76 (0.71–0.82)	0.043
	yes	0.48 (0.33–2.31)		0.49 (0.44–0.73)	

Data presented as medians with 95% CI and analyzed using Mann–Whitney U test or Kruskal–Wallis H test. AC, atherogenic coefficient; AIP, atherogenic index of plasma; BMI, body mass index; CHOL, cholesterol; CI, confidence interval; CRI, Castelli’s risk index; CRP, C-reactive protein; CV risk, cardiovascular risk; F, females; HS, hidradenitis suppurativa; IHS4, international hidradenitis suppurativa severity score system; IR, insulin resistance; LCI, lipoprotein combine index; M, males; N, number of cases; *p*, probability; TG, triacylglycerols; TyG, triglyceride-to-glucose index; ↑, elevated.

**Table 5:** Correlation of circulating gasdermins with selected demographical, anthropometrical, and clinical data.

Factor	Category	*N*	GSDMD		GSDME	
			ρ	*p* Value	ρ	*p* Value
Age	all	51	−0.09	0.522	0.0	0.992
IHS4	all	48	−0.12	0.427	0.02	0.883
HS duration	all	41	0.02	0.913	0.03	0.830
RBC	all	44	−0.35	0.020	0.11	0.521
Hb	all	44	−0.16	0.295	0.30	0.051
	F	20	0.24	0.302	0.14	0.543
	M	24	−0.21	0.316	0.47	0.019
BMI	all	43	−0.33	0.032	−0.04	0.819
	≥30	21	−0.43	0.051	0.24	0.296
GLU	F	15	−0.14	0.629	−0.59	0.020
	M	17	−0.20	0.450	0.47	0.058
TyG	F	15	0.36	0.186	−0.62	0.013
	M	16	−0.19	0.471	0.37	0.161
tCHOL	F	18	0.31	0.216	−0.50	0.034
	M	13	0.12	0.687	0.20	0.463
LDL-CHOL	F	17	0.04	0.885	−0.51	0.037
	M	13	0.10	0.734	−0.03	0.915
Non-HDL-CHOL	F	18	0.19	0.464	−0.45	0.061
	M	13	0.04	0.892	0.17	0.535
TG	F	18	0.46	0.058	−0.50	0.036
	M	16	−0.08	0.782	0.21	0.434
LCI	F	17	0.26	0.313	−0.55	0.032
	M	16	−0.02	0.936	0.21	0.441
AIP	F	17	0.36	0.155	−0.43	0.084
	M	16	−0.38	0.148	0.05	0.867
CREA	all	37	−0.03	0.872	0.24	0.015
AST	F	19	0.09	0.702	−0.45	0.056
ALP	F	16	−0.49	0.055	−0.29	0.283
Ferritin	F	14	0.58	0.029	−0.13	0.657
Fe	F	7	0.76	0.047	−0.13	0.782
	M	13	−0.07	0.825	0.52	0.071

Data were analyzed using Spearman rank correlation test and presented as rho (ρ) coefficients. AC, atherogenic coefficient; AIP, atherogenic index of plasma; ALP, alkaline phosphatase; AST, asparagine transaminase; BMI, body mass index; CHOL, cholesterol; CREA, creatinine; F, females; GLU, glucose; Hb, hemoglobin; IHS4, international hidradenitis suppurativa severity score system; LCI, lipoprotein combine index; M, males; *N*, number of cases; *p*, probability; ρ, Spearman’s rank correlation coefficient; RBC, red blood cell count; tCHOL, total cholesterol; TG, triacylglycerols; TyG, triglyceride-to-glucose index.

**Table 6:** Impact of selected patients’ and disease characteristics on *GSDMD* expression in the skin.

Factor	Cat.	Skin Expression of *GSDMD*					
		AIL	*p*	ANS	*p*	Fold Change	*p*
Sex	F	1.4 (0.9–2.3)	0.293	1.4 (0.6–3.2)	0.555	1.1 (0.5–2.2)	0.156
	M	1.9 (1.4–2.7)		1.1 (0.7–1.7)		1.8 (1.2–2.8)	
Hurley	2	1.7 (1.1–2.4)	0.374	1.1 (0.6–1.8)	0.858	1.6 (1–2.4)	0.407
	3	1.3 (0.9–2)		1.2 (0.4–3.3)		1.1 (0.5–2.7)	
IHS4	moderate	2.2 (0.8–6.2)	0.215	1.3 (0.2–8.2)	0.783	1.7 (0.4–7)	0.599
	severe	1.5 (1.1–1.9)		1.1 (0.7–1.9)		1.3 (0.9–2)	
Duration	<10 yrs.	1.6 (1.2–2)	0.322	1.1 (0.6–1.9)	0.683	1.5 (0.9–2.5)	0.890
	≥10 yrs.	2.1 (0.7–5.8)		1.3 (0.3–6.1)		1.6 (0.5–4.9)	
Smoking	no	2.0 (1.0–3.8)	0.407	1.0 (0.6–1.5)	0.801	2.1 (1.3–3.2)	0.342
	yes	1.6 (1.1–2.2)		1.1 (0.5–2.2)		1.5 (0.8–2.7)	
HyperUA	no	2.3 (1.6–3.3)	0.038	2.2 (0.9–5.2)	0.228	1.1 (0.5–2.5)	0.877
	yes	1.4 (0.9–2.2)		1.2 (0.5–2.8)		1.1 (0.5–2.7)	

Data were analyzed using *t*-test for independent samples and presented as geometric means of NRQ with 95% CI. AIL, actively inflamed lesions; ANS, patient-matched adjacent healthy-appearing skin; cat., category; CI, confidence interval; F, females; fold change, AIL-to-ANS ratio; HS, hidradenitis suppurativa; hyperUA, hyperuricemia; IHS4, international hidradenitis suppurativa severity score system; M, males; NRQ, normalized relative quantities; *p*, probability; yrs., years.

**Table 7:** Correlation of *GSDMD* mRNA level in the skin with selected demographical, anthropometrical, and clinical data of HS patients.

Factor	AIL		ANS		Fold Change	
	Corr. Coeff.	*p*	Corr. Coeff.	*p*	Corr. Coeff.	*p*
Age	*r* = 0.20	0.368	*r* = 0.08	0.716	*r* = 0.06	0.778
IHS4	*τ* = −0.24	0.141	*τ* = −0.26	0.106	*τ* = 0.14	0.408
HS duration	*τ* = 0.14	0.484	*τ* = 0.09	0.689	*τ* = 0.07	0.764
UA	*r* = −0.58	0.062	*r* = −0.41	0.214	*r* = 0.10	0.778
tBIL	*τ* = 0	0.955	*τ* = 0.37	0.077	*τ* = −0.37	0.060
GGT	*r* = 0.46	0.085	*r* = 0.20	0.478	*r* = 0.08	0.777

Data were analyzed using Pearson’s Product Moment Correlation test or Kendall rank correlation test and presented as *r* or tau (*τ*) coefficients. AIL, actively inflamed lesions; ANS, patient-matched adjacent healthy-appearing skin; corr. coeff., correlation coefficient; fold change, AIL-to-ANS ratio; GGT, gamma-glutamyl transferase; HS, hidradenitis suppurativa; IHS4, international hidradenitis suppurativa severity score system; *p*, probability; tBIL, total bilirubin; UA, uric acid.

**Table 8:** Impact of selected patients’ and disease characteristics on *GSDME* expression in the skin.

Factor	Cat.	Skin Expression of *GSDME*					
		AIL	*p*	ANS	*p*	Fold Change	*p*
Sex	F	1.6 (1.2–2.1)	0.284	1.0 (0.5–1.8)	0.244	1.7 (0.9–3.1)	0.478
	M	1.3 (1.1–1.7)		0.6 (0.4–1)		2.2 (1.4–3.4)	
Hurley	2	1.4 (1–1.9)	0.820	0.8 (0.5–1.4)	0.515	1.7 (1.1–2.8)	0.414
	3	1.5 (1.2–1.9)		0.6 (0.3–1.5)		2.4 (1.1–5.3)	
IHS4	moderate	1.5 (1–2.2)	0.944	0.6 (0–8.6)	0.708	2.4 (1.1–39)	0.660
	severe	1.4 (1.2–1.8)		0.8 (0.5–1.2)		1.9 (1.3–2.8)	
Duration	<10 yrs.	1.5 (1.3–1.8)	0.449	0.6 (0.3–1.2)	0.324	2.4 (1.3–4.4)	0.430
	≥10 yrs.	1.7 (0.9–3.3)		1.1 (0.4–3)		1.6 (0.4–5.5)	
Smoking	no	1.5 (1.1–2.1)	0.936	0.8 (0.3–2.2)	0.971	2 (0.8–5.4)	0.948
	yes	1.5 (1.3–1.8)		0.8 (0.5–1.2)		2 (1.2–3.1)	
IR (TyG)	no	1.8 (1.3–2.3)	0.255	0.6 (0.3–1.3)	0.163	3 (1.5–6)	0.055
	yes	1.5 (1.1–1.9)		1.2 (0.5–3.1)		1.2 (0.5–2.8)	
↓ HDL-C	no	1.8 (1.3–2.5)	0.099	0.8 (0.4–1.8)	0.822	2.3 (1–5.1)	0.736
	yes	1.4 (1.2–1.7)		0.7 (0.3–1.6)		1.9 (1–3.8)	
HyperTG	no	1.7 (1.3–2.1)	0.410	0.6 (0.3–1.1)	0.112	2.8 (1.7–4.7)	0.039
	yes	1.5 (1.1–1.9)		1.2 (0.5–3.1)		1.2 (0.5–2.8)	
↑AIP	no	1.5 (1.2–2)	0.432	0.5 (0.3–0.9)	0.027	2.8 (1.7–4.7)	0.049
	yes	1.7 (1.2–2.5)		1.4 (0.6–3.6)		1.2 (0.5–2.9)	
↑CRI2	no	1.6 (1.2–2)	0.641	0.7 (0.4–1.1)	0.056	2.3 (1.5–3.6)	0.078
	yes	1.7 (1.4–2.1)		1.7 (0.3–12)		1 (0.1–7.3)	

Data were analyzed using *t*-test for independent samples and presented as geometric means of NRQ with 95% CI. AIL, actively inflamed lesions; AIP, atherogenic index of plasma; ANS, patient-matched adjacent healthy-appearing skin; cat., category; CI, confidence interval; CRI, Castelli’s risk index; F, females; fold change, AIL-to-ANS ratio; HDL-C, high density lipoprotein cholesterol; HS, hidradenitis suppurativa; IHS4, international hidradenitis suppurativa severity score system; IR, insulin resistance; M, males; NRQ, normalized relative quantities; *p*, probability; TG, triacylglycerols; TyG, triglyceride-to-glucose index; yrs. years;↑, elevated; ↓, decreased.

**Table 9:** Correlation of *GSDME* mRNA level in the skin with selected demographical, anthropometrical, and clinical data of HS patients.

Factor	AIL		ANS		Fold Change	
	Corr. Coeff.	*p*	Corr. Coeff.	*p*	Corr. Coeff.	*p*
Age	*r* = 0.28	0.208	*r* = 0.62	0.002	*r* = −0.52	0.014
IHS4	*τ* = −0.06	0.693	*τ* = −0.28	0.091	*τ* = 0.26	0.122
HS duration	*τ* = 0.20	0.318	*τ* = 0.34	0.089	*τ* = −0.30	0.110
HOMA–IR	*τ* = 0.07	0.814	*τ* = 0.29	0.239	*τ* = −0.40	0.071
CRI-2	*τ* = −0.27	0.169	*τ* = 0.18	0.409	*τ* = −0.35	0.069
LCI	*τ* = −0.36	0.073	*τ* = 0.10	0.665	*τ* = −0.23	0.240
Ferritin	*r* = 0.05	0.885	*r* = 0.69	0.012	*r* = −0.71	0.010
IgA	*r* = −0.32	0.317	*r* = 0.45	0.147	*r* = −0.57	0.045

Data were analyzed using Pearson’s Product Moment Correlation test or Kendall rank correlation test and presented as *r* or tau (*τ*) coefficients. AIL, actively inflamed lesions; ANS, patient-matched adjacent healthy-appearing skin; corr. coeff., correlation coefficient; CRI, Castelli’s risk index; fold change, AIL-to-ANS ratio; HOMA-IR, homeostasis model assessment of insulin resistance; HS, hidradenitis suppurativa; IHS4, international hidradenitis suppurativa severity score system; LCI, lipoprotein combine index; *p*, probability.

## Data Availability

Data available on reasonable request from the corresponding author.

## Supplementary Materials

The following supporting information can be downloaded at: https://www.mdpi.com/article/10.3390/biology14091258/s1, Table S1: Impact of patients’ and disease characteristics on circulating gasdermins; Table S2: Correlation of circulating gasdermins with demographical, anthropometrical, and clinical data; Table S3: Impact of patients’ and disease characteristics on GSDMD expression in the skin; Table S4: Correlation of GSDMD mRNA level in the skin with demographical, anthropometrical, and clinical data of HS patients; Table S5: Impact of patients’ and disease characteristics on GSDME expression in the skin; Table S6: Correlation of GSDME mRNA level in the skin with demographical, anthropometrical, and clinical data of HS patients.
