# Supplementary material for: Gasdermin-Mediated Pyroptosis in Hidradenitis Suppurativa: Molecular Insights and Therapeutic Implications
Source: Biology (Basel). 2025 Sep 12;14(9):1258. doi: 10.3390/biology14091258 (PMC12467371; doi:10.3390/biology14091258)
Supplement: Supplementary file 1 [file biology-14-01258-s001.zip › biology-3587198-supplementary.pdf]

Supplementary material to:

Gasdermin-Mediated Pyroptosis in Hidradenitis Suppurativa: Molecular Insights and Therapeutic Implications

by Piotr K Krajewski, Kinga Tyczyńska, Aleksandra Sójka, Danuta Nowicka-Suszko, Iwona, Bednarz-Misa, Mariusz Fleszar, Małgorzata Krzystek-Korpacka and Jacek C Szepietowski

Supplementary Table S1. Impact of patients' and the disease characteristics on circulating gasdermins

Factor	Categories	GSDMD		GSDME	
		Median	*P* value	Median	*P* value
Sex	females	0.34 (0.25–0.54)	0.364	0.62 (0.5–0.73)	0.221
	males	0.31 (0.20–0.45)		0.72 (0.63–0.77)	
Hurley score	1/2	0.42 (0.32–0.67)	0.793	0.71 (0.61–0.77)	0.205
	3	0.34 (0.25–1.32)		0.62 (0.5–0.72)	
HS severity by IHS4	moderate	0.58 (0.32–1.56)	0.063	0.56 (0.46–0.79)	0.352
	severe	0.33 (0.26–0.54)		0.69 (0.62–0.76)	
Smoking habit	no	0.54 (0.31–1.49)	0.404	0.72 (0.47–0.76)	0.716
	yes	0.34 (0.27–0.59)		0.70 (0.59–0.77)	
	in the past	0.38 (0.19–0.80)		0.73 (0.57–0.84)	
BMI	normal	0.47 (0.35–1.13)	0.473	0.76 (0.64–0.82)	0.145
	overweight	0.52 (0.22–2.34)		0.62 (0.46–0.73)	
	obese	0.32 (0.25–0.68)		0.65 (0.5–0.78)	
BMI	normal	0.47 (0.35–1.13)	0.438	0.76 (0.64–0.82)	0.093
	elevated	0.34 (0.29–0.74)		0.65 (0.54–0.74)	
Anemia	no	0.39 (0.32–0.62)	0.811	0.72 (0.65–0.76)	0.005
	yes	0.38 (0.17–6.19)		0.48 (0.37–0.61)	
IFG	no	0.45 (0.32–1.14)	0.404	0.74 (0.67–0.77)	0.299
	yes	0.32 (0.13–1.16)		0.55 (0.46–0.92)	
HI	no	0.26 (0.07–1.45)	0.950	0.73 (0.6–0.79)	0.573
	yes	0.32 (0.1–0.61)		0.77 (0.46–0.96)	
IR (by HOMA-IR)	no	0.38 (0.09–1.31)	0.582	0.73 (0.6–0.78)	0.698
	yes	0.32 (0.3–1.28)		0.76 (0.6–0.83)	
IR (by TyG) F	no	0.36 (0.19–1.31)	0.594	0.76 (0.61–0.9)	0.007
	yes	0.98 (0.09–1.89)		0.48 (0.43–0.63)	
IR (by TyG) M	no	0.43 (0.06–0.96)	0.828	0.73 (0.44–0.79)	0.448
	yes	0.38 (0.15–3.4)		0.77 (0.63–0.79)	
HyperCHOL F	no	0.43 (0.2–1.08)	0.281	0.71 (0.6–0.79)	0.031
	yes	0.99 (0.3–1.92)		0.48 (0.43–0.85)	
HyperCHOL M	no	0.42 (0.17–0.84)	0.745	0.73 (0.65–0.79)	0.515
	yes	0.38 (0.13–7.42)		0.77 (0.52–0.97)	
↓ HDL-CHOL	no	0.47 (0.31–1.13)	0.899	0.70 (0.61–0.78)	0.884
	yes	0.41 (0.2–0.7)		0.73 (0.57–0.77)	
↑ LDL-CHOL F	no	0.36 (0.17–1.34)	0.546	0.72 (0.58–0.8)	0.063
	yes	0.74 (0.3–1.88)		0.52 (0.43–0.85)	
↑ LDL-CHOL M	no	0.48 (0.1–1.45)	1.0	0.71 (0.43–0.91)	0.475
	yes	0.45 (0.13–7.26)		0.76 (0.6–0.82)	
↑ nonHDL-CHOL F	no	0.34 (0.16–1.26)	0.230	0.73 (0.62–0.81)	0.029
	yes	0.76 (0.31–1.47)		0.54 (0.46–0.71)	

↑ nonHDL-CHOL M	no	0.48 (0.25–1.2)	0.692	0.73 (0.56–0.8)	0.396
	yes	0.38 (0.14–1.72)		0.76 (0.68–0.81)	
HyperTG F	no	0.41 (0.23–1.26)	0.450	0.73 (0.59–0.81)	0.050
	yes	0.76 (0.27–1.47)		0.54 (0.46–0.72)	
HyperTG M	no	0.43 (0.07–0.99)	0.828	0.73 (0.45–0.79)	0.448
	yes	0.38 (0.16–4.4)		0.77 (0.63–0.9)	
↑ CV risk by CRI-1 F	no	0.34 (0.28–1.13)	0.068	0.71 (0.48–0.79)	0.283
	yes	1.34 (0.8–1.89)		0.58 (0.52–0.59)	
↑ CV risk by CRI-1 M	no	0.48 (0.1–1.48)	0.560	0.73 (0.62–0.79)	0.491
	yes	0.32 (0.16–4.02)		0.76 (0.57–0.79)	
↑ CV risk by CRI-2 F	no	0.36 (0.29–1.16)	0.179	0.7 (0.49–0.78)	0.502
	yes	1.34 (0.62–2.06)		0.59 (0.58–0.59)	
↑ CV risk by CRI-2 M	no	0.47 (0.1–1.17)	0.865	0.75 (0.67–0.81)	0.865
	yes	0.38 (0.29–2.22)		0.73 (0.61–0.83)	
↑ CV risk by AC F	no	0.32 (0.24–1.0)	0.047	0.72 (0.54–0.81)	0.089
	yes	1.23 (0.87–1.7)		0.54 (0.48–0.59)	
↑ CV risk by AC M	no	0.59 (0.07–1.3)	0.628	0.74 (0.67–0.76)	0.716
	yes	0.41 (0.23–0.55)		0.75 (0.66–0.83)	
↑ CV risk by AIP F	no	0.36 (0.29–1.05)	0.044	0.7 (0.51–0.78)	0.296
	yes	1.7 (1.34–2.06)		0.54 (0.5–0.58)	
↑ CV risk by AIP M	no	0.52 (0.14–2.57)	0.248	0.74 (0.57–0.81)	0.834
	yes	0.32 (0.15–0.43)		0.75 (0.62–0.96)	
↑ CV risk by LCI F	no	0.32 (0.23–0.84)	0.036	0.72 (0.50–0.81)	0.023
	yes	1.23 (0.98–1.66)		0.48 (0.46–0.54)	
↑ CV risk by LCI M	no	0.48 (0.06–1.3)	0.791	0.73 (0.49–0.79)	0.368
	yes	0.38 (0.22–4.81)		0.76 (0.66–0.93)	
HyperUA F	no	0.56 (0.32–1.25)	0.903	0.64 (0.47–0.81)	0.668
	yes	0.67 (0.26–1.06)		0.67 (0.55–0.73)	
HyperUA M	no	0.52 (0.08–7.08)	0.165	0.72 (0.47–0.79)	0.203
	yes	0.31 (0.13–0.42)		0.76 (0.67–0.97)	
Leukocytosis	no	0.39 (0.3–0.92)	0.946	0.7 (0.6–0.76)	0.515
	yes	0.32 (0.29–0.96)		0.65 (0.49–0.74)	
Neutrophilia	no	0.39 (0.3–0.88)	0.864	0.69 (0.61–0.76)	0.171
	yes	0.4 (0.3–0.61)		0.62 (0.4–0.72)	
↑ NLR	no	0.38 (0.29–0.94)	0.959	0.69 (0.62–0.77)	0.246
	yes	0.38 (0.29–0.61)		0.61 (0.45–0.74)	
Inflammation by CRP F	no	0.67 (0.32–1.65)	0.310	0.67 (0.46–0.78)	0.536
	yes	0.36 (0.17–1.31)		0.65 (0.51–0.89)	
Inflammation by CRP M	no	0.32 (0.13–0.76)	0.218	0.76 (0.71–0.82)	0.043
	yes	0.48 (0.33–2.31)		0.49 (0.44–0.73)	

Data presented as medians with 95%CI and analyzed using Mann-Whitney U test or Kruskal-Wallis H test. AC, atherogenic coefficient; AIP, atherogenic index of plasma; BMI, body mass index; CHOL, cholesterol; CI, confidence interval; CRI, Castelli's risk index; CRP, C-reactive protein; CV risk, cardiovascular risk; F, females; HI, hyperinsulinemia; HOMA-IR, homeostasis model assessment of insulin resistance; HS, hidradenitis suppurativa; IFG, impaired fasting glucose; IHS4, international hidradenitis suppurativa severity score system; IR, insulin resistance; LCI, lipoprotein combine index; M, males; N, number of cases; NLR, neutrophil-to-lymphocyte ratio; *p*, probability; TG, triacylglycerols; TyG, triglyceride-to-glucose index; UA, uric acid; ↑, elevated; ↓, decreased.

Supplementary Table S2. Correlation of circulating gasdermins with demographical, anthropometrical and clinical data

Factor	Category	*N*	GSDMD		GSDME	
			ϱ	*P* value	ϱ	*P* value
Age	all	51	-0.09	0.522	0.0	0.992
IHS4	all	48	-0.12	0.427	0.02	0.883
HS duration	all	41	0.02	0.913	0.03	0.830
RBC	all	44	-0.35	0.020	0.11	0.521
Hb	all	44	-0.16	0.295	0.30	0.051
	F	20	0.24	0.302	0.14	0.543
	M	24	-0.21	0.316	0.47	0.019
BMI	all	43	-0.33	0.032	-0.04	0.819
	≥ 30	21	-0.43	0.051	0.24	0.296
GLU	all	32	-10	0.580	-0.07	0.720
	F	15	-0.14	0.629	-0.59	0.020
	M	17	-0.20	0.450	0.47	0.058
INS	all	24	-0.04	0.875	0.16	0.941
	F	10	0.44	0.200	-0.46	0.179
	M	14	-0.26	0.375	0.35	0.221
HOMA–IR	all	22	-0.08	0.732	0.07	0.756
	F	8	0.32	0.435	-0.54	0.168
	M	14	-0.21	0.469	0.34	0.230
TyG	all	31	0.10	0.586	-0.07	0.727
	F	15	0.36	0.186	-0.62	0.013
	M	16	-0.19	0.471	0.37	0.161
tCHOL	all	34	0.18	0.304	-0.08	0.657
	F	18	0.31	0.216	-0.50	0.034
	M	13	0.12	0.687	0.20	0.463
LDL-CHOL	all	30	0.12	0.517	-0.18	0.350
	F	17	0.04	0.885	-0.51	0.037
	M	13	0.10	0.734	-0.03	0.915
HDL-CHOL	all	33	0.03	0.858	-0.03	0.892
	F	17	0.01	0.983	0.08	0.764
	M	16	-0.13	0.643	0.01	0.978
nonHDL-CHOL	all	34	0.15	0.403	-0.06	0.743
	F	18	0.19	0.464	-0.45	0.061
	M	13	0.04	0.892	0.17	0.535
TG	all	34	0.19	0.296	-0.10	0.576
	F	18	0.46	0.058	-0.50	0.036
	M	16	-0.08	0.782	0.21	0.434
CRI-1	all	33	0.02	0.911	-0.01	0.974
CRI-2	all	33	0.05	0.802	-0.05	0.765
AC	all	33	0.02	0.911	-0.01	0.974
LCI	all	33	0.15	0.391	-0.09	0.615
	F	17	0.26	0.313	-0.55	0.032
	M	16	-0.02	0.936	0.21	0.441
AIP	all	33	-0.04	0.843	-0.06	0.748
	F	17	0.36	0.155	-0.43	0.084

	M	16	-0.38	0.148	0.05	0.867
CREA	all	37	-0.03	0.872	0.24	0.015
Urea	all	32	-0.19	0.307	0.29	0.109
UA	all	30	-0.20	0.279	0.07	0.719
tBIL	all	34	0.07	0.680	-0.03	0.851
ALT	all	38	-0.01	0.972	-0.08	0.651
AST	all	38	0.01	0.933	-0.2	0.229
	F	19	0.09	0.702	-0.45	0.056
	M	19	-0.12	0.636	0.01	0.954
GGT	all	38	0.07	0.684	-0.04	0.819
ALP	all	30	-0.28	0.138	-0.13	0.481
	F	16	-0.49	0.055	-0.29	0.283
	M	14	0.11	0.714	-0.09	0.759
CRP	all	36	-0.10	0.576	-0.06	0.748
WBC	all	44	-0.17	0.284	0.01	0.938
NEU	all	44	0.11	0.474	0.00	0.935
LYM	all	44	-0.04	0.814	0.18	0.238
PLT	all	44	0.01	0.958	-0.02	0.881
NLR	all	44	0.00	0.995	-0.02	0.906
PLR	all	44	0.12	0.434	-0.18	0.261
SII	all	44	0.01	0.939	-0.05	0.691
Ferritin	F	14	0.58	0.029	-0.13	0.657
	M	16	0.27	0.316	0	0.994
Fe	F	7	0.76	0.047	-0.13	0.782
	M	13	-0.07	0.825	0.52	0.071
IgA	all	29	-0.03	0.887	0.10	0.619

Data were analyzed using Spearman rank correlation test and presented as rho (ϱ) coefficients.

AC, atherogenic coefficient; AIP, atherogenic index of plasma; ALP, alkaline phosphatase; ALT, alanine transaminase; AST, asparagine transaminase; BMI, body mass index; CHOL, cholesterol; CREA, creatinine; CRI, Castelli's risk index; CRP, C-reactive protein; F, females; Fe, iron; GGT, gamma-glutamyl transferase; GLU, glucose; Hb, hemoglobin; HOMA-IR, homeostasis model assessment of insulin resistance; IHS4, international hidradenitis suppurativa severity score system; INS, insulin; LCI, lipoprotein combine index; LYM, lymphocyte count; M, males; N, number of cases; NEU, neutrophil count; NLR, neutrophil-to-lymphocyte ratio; *p*, probability; PLR, platelet-to-lymphocyte ratio; PLT, platelet count; ϱ, Spearman's rank correlation coefficient; SII, systemic immune-inflammation index; RBC, red blood cells count; tBIL, total bilirubin; tCHOL, total cholesterol; TG, triacylglycerols; TyG, triglyceride-to-glucose index; UA, uric acid; WBC, leukocyte count.

Supplementary Table S3. Impact of patients′ and the disease characteristics on *GSDMD* expression in the skin

Factor	Cat.	Skin expression of *GSDMD*					
		AIL	*P*	ANS	*P*	Fold-change	*P*
Sex	F	1.4 (0.9–2.3)	0.293	1.4 (0.6–3.2)	0.555	1.1 (0.5–2.2)	0.156
	M	1.9 (1.4–2.7)		1.1 (0.7–1.7)		1.8 (1.2–2.8)	
Hurley	2	1.7 (1.1–2.4)	0.374	1.1 (0.6–1.8)	0.858	1.6 (1–2.4)	0.407
	3	1.3 (0.9–2)		1.2 (0.4–3.3)		1.1 (0.5–2.7)	
IHS4	moderate	2.2 (0.8–6.2)	0.215	1.3 (0.2–8.2)	0.783	1.7 (0.4–7)	0.599
	severe	1.5 (1.1–1.9)		1.1 (0.7–1.9)		1.3 (0.9–2)	
Duration	<10	1.6 (1.2–2)	0.322	1.1 (0.6–1.9)	0.683	1.5 (0.9–2.5)	0.890
	≥10	2.1 (0.7–5.8)		1.3 (0.3–6.1)		1.6 (0.5–4.9)	
Smoking	no	2.0 (1.0–3.8)	0.407	1.0 (0.6–1.5)	0.801	2.1 (1.3–3.2)	0.342
	yes	1.6 (1.1–2.2)		1.1 (0.5–2.2)		1.5 (0.8–2.7)	
↑BMI	no	1.7 (0.9–3.4)	0.935	1.2 (0.5–2.6)	0.923	1.4 (0.6–3.5)	0.946
	yes	1.7 (1.3–2.3)		1.2 (0.7–2.3)		1.4 (0.8–2.3)	
IFG	no	1.9 (1.4–2.6)	0.213	1.9 (1.1–3.2)	0.327	1 (0.6–1.7)	0.594
	yes	1.4 (0.7–3)		1.1 (0.2–7.2)		1.3 (0.4–4.8)	
IR (TyG)	no	1.7 (1.1–2.7)	0.792	1.5 (0.7–3.5)	0.953	1.1 (0.6–1.9)	0.932
	yes	1.8 (1.3–2.6)		1.6 (0.5–5)		1.1 (0.4–3)	
Inflammation	no	1.8 (1.3–2.4)	0.950	1.5 (0.8–2.8)	0.717	1.2 (0.7–2)	0.707
	yes	1.8 (1–3)		1.3 (0.6–3)		1.3 (0.7–2.7)	
leukocytosis	no	1.3 (0.9–1.9)	0.337	0.9 (0.4–1.6)	0.208	1.6 (0.9–2.6)	0.462
	yes	1.8 (1–3.1)		1.5 (0.8–2.7)		1.2 (0.6–2.3)	
neutrophilia	no	1.4 (1.1–1.9)	0.370	1.1 (0.6–1.8)	0.951	1.3 (0.9–2)	0.521
	yes	2 (0.3–12.2)		1.1 (0.8–1.4)		1.8 (0.3–12.3)	
↑NLR	no	1.6 (1.1–2.2)	0.733	1.2 (0.7–2)	0.514	1.3 (0.9–1.9)	0.612
	yes	1.4 (0.8–2.6)		0.9 (0.3–2.4)		1.6 (0.7–3.8)	
hyperCHOL	no	1.7 (1.1–2.5)	0.865	1.3 (0.7–2.5)	0.868	1.3 (0.7–2.2)	0.759
	yes	1.6 (0.9–2.9)		1.5 (0.4–5.2)		1.1 (0.47–2.8)	
↑LDL-C	no	1.7 (1.2–2.4)	0.869	1.5 (0.8–2.9)	0.892	1.1 (0.7–1.9)	0.954
	yes	1.6 (0.8–3.2)		1.4 (0.4–5.1)		1.1 (0.4–3.2)	
↑nonHDL-C	no	1.6 (1–2.7)	0.912	1.6 (0.7–3.6)	0.637	1 (0.5–2.1)	0.526
	yes	1.7 (1.1–2.6)		1.3 (0.6–2.8)		1.3 (0.7–2.6)	
↓HDL-C	no	1.8 (1.1–3.1)	0.726	1.9 (0.7–5.3)	0.343	1 (0.5–2)	0.364
	yes	1.6 (1.2–2.3)		1.2 (0.7–2.1)		1.4 (0.8–2.3)	
hyperTG	no	1.7 (1.1–2.5)	0.745	1.4 (0.8–2.6)	0.806	1.2 (0.7–1.9)	0.936
	yes	1.8 (1.3–2.6)		1.6 (0.5–5)		1.1 (0.4–3)	
↑AIP	no	1.6 (1.1–2.5)	0.679	1.6 (0.8–3.2)	0.496	1 (0.6–1.6)	0.232
	yes	1.8 (1.2–2.7)		1.2 (0.5–2.8)		1.5 (0.6–3.8)	
↑AC	no	1.5 (0.8–2.7)	0.434	1.5 (0.6–3.7)	0.825	1 (0.5–2)	0.439
	yes	1.8 (1.3–2.5)		1.3 (0.6–2.8)		1.4 (0.7–2.7)	
↑CRI1	no	1.6 (1–2.4)	0.528	1.6 (0.7–3.3	0.526	1 (0.6–1.7)	0.234
	yes	1.8 (1.2–2.7)		1.1 (0.5–2.7)		1.6 (0.6–4.1)	
↑CRI2	no	1.5 (1.1–2.1)	0.239	1.3 (0.7–2.5)	0.720	1.2 (0.7–1.8)	0.749
	yes	2.2 (1–4.7)		1.6 (0.3–9.5)		1.3 (0.1–16.6)	
hyperUA	no	2.3 (1.6–3.3)	0.038	2.2 (0.9–5.2)	0.228	1.1 (0.5–2.5)	0.877
	yes	1.4 (0.9–2.2)		1.2 (0.5–2.8)		1.1 (0.5–2.7)	

Data were analyzed using t-test for independent samples and presented as geometric means of NRQ with 95% CI. AC, atherogenic coefficient; AIP, atherogenic index of plasma; ALP, alkaline phosphatase; ALT, alanine transaminase; AST, asparagine transaminase; BMI, body mass index; CHOL, cholesterol; CI, confidence interval; CREA, creatinine; CRI, Castelli's risk index; CRP, C-reactive protein; CV risk, cardiovascular risk; GGT, gamma-glutamyl transferase; HI, hyperinsulinemia; HOMA-IR, homeo-stasis model assessment of insulin resistance; HS, hidradenitis suppurativa; IFG, impaired fasting glucose; IHS4, international hidradenitis suppurativa severity score system; IR, insulin resistance; LCI, lipoprotein combine index; LYM, lymphocyte count; N, number of cases; NEU, neutrophil count; NLR, neutrophil-to-lymphocyte ratio; p, probability; PLR, platelet-to-lymphocyte ratio; PLT, platelet count; SII, systemic immune-inflammation index; tBIL, total bilirubin; tCHOL, total cho-lesterol; TG, triacylglycerols; TyG, triglyceride-to-glucose index; UA, uric acid; WBC, leukocyte count; yrs., years; ↑, elevated; ↓, decreased.

Supplementary Table S4. Correlation of *GSDMD* mRNA level in the skin with demographical, anthropometrical and clinical data of HS patients

Factor	AIL		ANS		Fold-change*	
	Corr. coeff.	*P*	Corr. coeff.	*P*	Corr. coeff.	*P*
Age	*r* = 0.20	0.368	*r* = 0.08	0.716	*r* = 0.06	0.778
IHS4	τ = -0.24	0.141	τ = -0.26	0.106	τ = 0.14	0.408
HS duration	τ = 0.14	0.484	τ = 0.09	0.689	τ = 0.07	0.764
Hb	*r* = 0.12	0.659	*r* = -0.06	0.828	*r* = 0.15	0.553
RBC	*r* = 0.12	0.644	*r* = -0.14	0.589	*r* = 0.26	0.317
BMI	τ = -0.14	0.401	τ = -0.08	0.615	τ = 0.01	1.0
GLU	*r* = -0.28	0.370	*r* = -0.28	0.380	*r* = 0.19	0.546
INS	*r* = -0.03	0.925	*r* = -0.32	0.286	*r* = 0.37	0.218
HOMA–IR	τ = -0.15	0.480	τ = -0.18	0.388	τ = 0.26	0.307
TyG	τ = -0.14	0.490	τ = -0.11	0.580	τ = 0.05	0.890
tCHOL	*r* = 0.07	0.828	*r* = 0.12	0.695	*r* = -0.10	0.748
LDL-CHOL	*r* = -0.09	0.772	*r* = 0.09	0.764	*r* = -0.17	0.569
HDL-CHOL	*r* = -0.06	0.847	*r* = 0.18	0.540	*r* = -0.26	0.379
nonHDL-CHOL	*r* = 0.02	0.945	*r* = 0.02	0.951	*r* = -0.01	0.978
TG	τ = -0.03	0.827	τ = -0.01	0.913	τ = -0.03	0.827
CRI-1	τ = 0.08	0.760	τ = -0.15	0.428	τ = 0.10	0.669
CRI-2	τ = 0.04	0.869	τ = -0.02	0.869	τ = 0.04	0.867
AC	τ = 0.08	0.760	τ = -0.15	0.428	τ = 0.10	0.669
LCI	τ = 0.03	0.951	τ = -0.16	0.422	τ = 0.08	0.757
AIP	τ = 0.08	0.743	τ = -0.12	0.511	τ = 0.25	0.228
CREA	*r* = -0.06	0.831	*r* = 0.11	0.672	*r* = -0.17	0.521
Urea	*r* = -0.35	0.248	*r* = -0.01	0.964	*r* = -0.21	0.487
UA	*r* = -0.58	0.062	*r* = -0.41	0.214	*r* = 0.10	0.778
tBIL	τ = 0	0.955	τ = 0.37	0.077	τ = -0.37	0.060
GGT	*r* = 0.46	0.085	*r* = 0.20	0.478	*r* = 0.08	0.777
AST	*r* = 0.17	0.520	*r* = 0.25	0.353	*r* = -0.20	0.462
ALT	*r* = 0.35	0.205	*r* = 0.23	0.409	*r* = -0.04	0.882
ALP	*r* = 0.38	0.150	*r* = 0.22	0.418	*r* = 0.19	0.596
CRP	*r* = 0.36	0.185	*r* = -0.13	0.641	*r* = 0.40	0.145
WBC	*r* = 0.31	0.231	*r* = -0.05	0.836	*r* = 0.30	0.247
NEU	*r* = 0.33	0.193	*r* = -0.03	0.911	*r* = 0.29	0.265
LYM	*r* = 0.32	0.222	*r* = -0.13	0.642	*r* = 0.39	0.134
PLT	*r* = 0.23	0.366	*r* = -0.13	0.609	*r* = 0.34	0.188
NLR	τ = 0.12	0.534	τ = 0.04	0.836	τ = 0.07	0.709
PLR	τ = -0.08	0.620	τ = 0.07	0.741	τ = -0.10	0.563
SII	τ = 0.19	0.303	τ = 0.03	0.902	τ = 0.19	0.537
Ferritin	*r* = 0.37	0.235	*r* = 0.07	0.829	*r* = 0.17	0.604
Fe	*r* = 0.06	0.883	*r* = 0.01	0.975	*r* = -0.17	0.663
IgA	*r* = 0.39	0.216	*r* = 0.41	0.181	*r* = -0.23	0.472

Data were analyzed using Pearson's Product Moment Correlation test or Kendall rank correlation test and presented as *r* or tau (τ) coefficients.

AC, atherogenic coefficient; AIP, atherogenic index of plasma; ALP, alkaline phosphatase; ALT, alanine transaminase; AST, asparagine transaminase; BMI, body mass index; CHOL, cholesterol; CREA, creatinine; CRI, Castelli's risk index; CRP, C-reactive protein; F, females; Fe, iron; GGT, gamma-glutamyl transferase; GLU, glucose; Hb, hemoglobin; HOMA-IR, homeostasis model assessment of insulin resistance; IHS4, international hidradenitis suppurativa severity score system; INS, insulin; LCI, lipoprotein combine index; LYM, lymphocyte count; M, males; N, number of cases; NEU, neutrophil count; NLR, neutrophil-to-lymphocyte ratio; *p*, probability; PLR, platelet-to-lymphocyte ratio; PLT, platelet count; ϱ, Spearman's rank correlation coefficient; SII, systemic immune-inflammation index; RBC, red blood cells count; tBIL, total bilirubin; tCHOL, total cholesterol; TG, triacylglycerols; TyG, triglyceride-to-glucose index; UA, uric acid; WBC, leukocyte count.

Supplementary Table S5. Impact of patients' and the disease characteristics on *GSDME* expression in the skin

Factor	Cat.	Skin expression of *GSDME*					
		**AIL**	***P***	**ANS**	***P***	**Fold-change**	***P***
Sex	F	1.6 (1.2–2.1)	0.284	1.0 (0.5–1.8)	0.244	1.7 (0.9–3.1)	0.478
	M	1.3 (1.1–1.7)		0.6 (0.4–1)		2.2 (1.4–3.4)	
Hurley	2	1.4 (1–1.9)	0.820	0.8 (0.5–1.4)	0.515	1.7 (1.1–2.8)	0.414
	3	1.5 (1.2–1.9)		0.6 (0.3–1.5)		2.4 (1.1–5.3)	
IHS4	moderate	1.5 (1–2.2)	0.944	0.6 (0–8.6)	0.708	2.4 (1.1–39)	0.660
	severe	1.4 (1.2–1.8)		0.8 (0.5–1.2)		1.9 (1.3–2.8)	
Duration	<10	1.5 (1.3–1.8)	0.449	0.6 (0.3–1.2)	0.324	2.4 (1.3–4.4)	0.430
	≥10	1.7 (0.9–3.3)		1.1 (0.4–3)		1.6 (0.4–5.5)	
Smoking	no	1.5 (1.1–2.1)	0.936	0.8 (0.3–2.2)	0.971	2 (0.8–5.4)	0.948
	yes	1.5 (1.3–1.8)		0.8 (0.5–1.2)		2 (1.2–3.1)	
↑BMI	no	1.7 (1.1–2.6)	0.435	0.6 (1.1–2.3)	0.555	2.9 (0.8–10)	0.368
	yes	1.5 (1.3–1.8)		0.8 (0.5–1.3)		1.9 (1.2–3.1)	
IFG	no	1.6 (1.3–2.1)	0.955	0.9 (0.4–2.1)	0.612	1.6 (0.7–3.6)	0.611
	yes	1.6 (1.1–2.5)		0.7 (0.4–1)		2.5 (1.1–5.7)	
IR (TyG)	no	0.6 (0.3–1.3)	0.163	0.7 (0.3–1.7)	0.271	3 (1.5–6)	0.055
	yes	1.2 (0.5–3.1)		1.2 (0.5–3.1)		1.2 (0.5–2.8)	
Inflammation	no	1.6 (1.1–2.1)	0.422	1.1 (0.4–1.9)	0.647	1.7 (0.8–3.6)	0.545
	yes	1.4 (1.1–1.7)		0.9 (0.3–1.5)		2.2 (1.1–4.4)	
leukocytosis	no	1.5 (1–2.1)	0.829	0.7 (0.3–1.5)	0.638	2 (1–4)	0.860
	yes	1.4 (1.1–1.8)		0.9 (0.5–1.7)		1.9 (0.9–3.8)	
neutrophilia	no	1.5 (1.1–1.9)	0.782	0.7 (0.4–1.2)	0.728	2.1 (1.2–3.4)	0.610
	yes	1.3 (0.7–2.7)		0.9 (0.2–4.9)		1.5 (0.3–8.9)	
↑NLR	no	1.5 (1–2.1)	0.821	0.8 (0.4–1.5)	0.825	1.9 (1–3.6)	0.896
	yes	1.4 (1.1–1.8)		0.7 (0.3–1.5)		2 (0.9–4.5)	
hyperCHOL	no	1.7 (1.3–2.2)	0.645	0.7 (0.4–1.2)	0.207	2.4 (1.5–4)	0.127
	yes	1.5 (1.1–2.2)		1.2 (0.4–3.2)		1.3 (0.5–3.5)	
↑LDL-C	no	1.6 (1.3–2.1)	0.821	0.7 (0.3–1.3)	0.664	2.5 (1.4–4.5)	0.560
	yes	1.6 (1.1–2.3)		0.8 (0.3–2.4)		1.9 (0.7–5.6)	
↑nonHDL-C	no	1.8 (1.3–2.4)	0.364	0.8 (0.3–1.7)	0.653	2.3 (1.1–4.8)	0.405
	yes	1.5 (1.2–1.9)		0.9 (0.4–2)		1.6 (0.8–3.3)	
↓HDL-C	no	1.8 (1.3–2.5)	0.099	0.8 (0.4–1.8)	0.822	2.3 (1–5.1)	0.736
	yes	1.4 (1.2–1.7)		0.7 (0.3–1.6)		1.9 (1–3.8)	
hyperTG	no	1.7 (1.3–2.1)	0.410	0.6 (0.3–1.1)	0.112	2.8 (1.7–4.7)	0.039
	yes	1.5 (1.1–1.9)		1.2 (0.5–3.1)		1.2 (0.5–2.8)	
↑AIP	no	1.5 (1.2–2)	0.432	0.5 (0.3–0.9)	0.027	2.8 (1.7–4.7)	0.049
	yes	1.7 (1.2–2.5)		1.4 (0.6–3.6)		1.2 (0.5–2.9)	
↑AC	no	1.8 (1.3–2.6)	0.167	0.7 (0.3–1.7)	0.591	2.5 (1.1–5.3)	0.265
	yes	1.5 (1.2–1.8)		0.9 (0.5–2)		1.5 (0.8–3)	
↑CRI1	no	1.6 (1.2–2.2)	0.992	0.7 (0.4–1.2	0.212	2.4 (1.3–4.2)	0.191
	yes	1.6 (1.3–2)		1.2 (0.5–3.1)		1.4 (0.5–3.5)	
↑CRI2	no	1.6 (1.2–2)	0.641	0.7 (0.4–1.1)	0.056	2.3 (1.5–3.6)	0.078
	yes	1.7 (1.4–2.1)		1.7 (0.3–12)		1 (0.1–7.3)	
hyperUA	no	1.7 (1.2–2.3)	0.370	0.9 (0.4–1.9)	0.902	2 (1–3.8)	0.618

Data were analyzed using t-test for independent samples and presented as geometric means of NRQ with 95% CI. AC, atherogenic coefficient; AIP, atherogenic index of plasma; ALP, alkaline phosphatase; ALT, alanine transaminase; AST, asparagine transaminase; BMI, body mass index; CHOL, cholesterol; CI, confidence interval; CREA, creatinine; CRI, Castelli's risk index; CRP, C-reactive protein; CV risk, cardiovascular risk; GGT, gamma-glutamyl transferase; HI, hyperinsulinemia; HOMA-IR, homeo-stasis model assessment of insulin resistance; HS, hidradenitis suppurativa; IFG, impaired fasting glucose; IHS4, international hidradenitis suppurativa severity score system; IR, insulin resistance; LCI, lipoprotein combine index; LYM, lymphocyte count; N, number of cases; NEU, neutrophil count; NLR, neutrophil-to-lymphocyte ratio; p, probability; PLR, platelet-to-lymphocyte ratio; PLT, platelet count; SII, systemic immune-inflammation index; tBIL, total bilirubin; tCHOL, total cho-lesterol; TG, triacylglycerols; TyG, triglyceride-to-glucose index; UA, uric acid; WBC, leukocyte count; yrs., years; ↑, elevated; ↓, decreased.

Supplementary Table S6. Correlation of *GSDME* mRNA level in the skin with demographical, anthropometrical and clinical data of HS patients

Factor	AIL		ANS		Fold-change*	
	Corr. coeff.	*P*	Corr. coeff.	*P*	Corr. coeff.	*P*
Age	*r* = 0.28	0.208	*r* = 0.62	0.002	*r* = -0.52	0.014
IHS4	τ = -0.06	0.693	τ = -0.28	0.091	τ = 0.26	0.122
HS duration	τ = 0.20	0.318	τ = 0.34	0.089	τ = -0.30	0.110
Hb	*r* = -0.18	0.487	*r* = 0.09	0.722	*r* = -0.19	0.472
RBC	*r* = -0.32	0.217	*r* = -0.27	0.304	*r* = 0.14	0.602
BMI	τ = -0.04	0.801	τ = 0.10	0.615	τ = -0.05	0.737
GLU	*r* = 0.14	0.653	*r* = 0.16	0.642	*r* = -0.12	0.736
INS	*r* = 0.12	0.697	*r* = 0.19	0.526	*r* = -0.16	0.593
HOMA–IR	τ = 0.07	0.814	τ = 0.29	0.239	τ = -0.40	0.071
TyG	τ = -0.23	0.269	τ = 0.08	0.782	τ = -0.23	0.268
tCHOL	*r* = -0.37	0.214	*r* = 0.30	0.326	*r* = -0.45	0.119
LDL-CHOL	*r* = -0.33	0.273	*r* = 0.31	0.299	*r* = -0.46	0.111
HDL-CHOL	*r* = 0.42	0.136	*r* = 0.17	0.568	*r* = -0.02	0.939
nonHDL-CHOL	*r* = -0.47	0.105	*r* = 0.25	0.419	*r* = -0.44	0.130
TG	τ = -0.32	0.100	τ = 0.06	0.827	τ = -0.14	0.443
CRI-1	τ = -0.26	0.200	τ = 0.13	0.583	τ = -0.26	0.200
CRI-2	τ = -0.27	0.169	τ = 0.18	0.409	τ = -0.35	0.069
AC	τ = -0.26	0.200	τ = 0.13	0.583	τ = -0.26	0.200
LCI	τ = -0.36	0.073	τ = 0.10	0.665	τ = -0.23	0.240
AIP	τ = -0.12	0.511	τ = 0.08	0.743	τ = -0.17	0.381
CREA	*r* = -0.12	0.667	*r* = -0.07	0.810	*r* = 0.03	0.911
Urea	*r* = 0.43	0.145	*r* = 0.28	0.350	*r* = -0.15	0.625
UA	*r* = 0.21	0.530	*r* = -0.11	0.755	*r* = 0.04	0.901
tBIL	τ = 0.02	0.955	τ = 0.07	0.776	τ = 0.05	0.864
GGT	*r* = -0.15	0.600	*r* = 0.09	0.743	*r* = -0.15	0.602
AST	*r* = 0.08	0.766	*r* = 0.20	0.456	*r* = -0.19	0.488
ALT	*r* = -0.13	0.633	*r* = 0.10	0.727	*r* = -0.15	0.592
ALP	*r* = -0.45	0.163	*r* = -0.11	0.746	*r* = -0.08	0.816
CRP	*r* = -0.20	0.482	*r* = -0.37	0.174	*r* = 0.31	0.259
WBC	*r* = 0.13	0.631	*r* = -0.11	0.662	*r* = 0.18	0.481
NEU	*r* = 0.18	0.489	*r* = -0.11	0.666	*r* = 0.21	0.423
LYM	*r* = 0.03	0.897	*r* = -0.19	0.460	*r* = 0.22	0.387
PLT	*r* = -0.13	0.623	*r* = -0.35	0.170	*r* = 0.32	0.316
NLR	τ = -0.07	0.648	τ = -0.06	0.709	τ = 0.07	0.709
PLR	τ = -0.16	0.363	τ = -0.14	0.408	τ = 0.05	0.804
SII	τ = -0.16	0.343	τ = -0.06	0.711	τ = 0.10	0.592
Ferritin	*r* = 0.05	0.885	*r* = 0.69	0.012	*r* = -0.71	0.010
Fe	*r* = 0.10	0.807	*r* = 0.56	0.119	*r* = -0.11	0.785
IgA	*r* = -0.32	0.317	*r* = 0.45	0.147	*r* = -0.57	0.045

ata were analyzed using Pearson's Product Moment Correlation test or Kendall rank correlation test and presented as *r* or tau (τ) coefficients.

AC, atherogenic coefficient; AIP, atherogenic index of plasma; ALP, alkaline phosphatase; ALT, alanine transaminase; AST, asparagine transaminase; BMI, body mass index; CHOL, cholesterol; CREA, creatinine; CRI, Castelli's risk index; CRP, C-reactive protein; F, females; Fe, iron; GGT, gamma-glutamyl transferase; GLU, glucose; Hb, hemoglobin; HOMA-IR, homeostasis model assessment of insulin resistance; IHS4, international hidradenitis suppurativa severity score system; INS, insulin; LCI, lipoprotein combine index; LYM, lymphocyte count; M, males; N, number of cases; NEU, neutrophil count; NLR, neutrophil-to-lymphocyte ratio; *p*, probability; PLR, platelet-to-lymphocyte ratio; PLT, platelet count; ϱ, Spearman's rank correlation coefficient; SII, systemic immune-inflammation index; RBC, red blood cells count; tBIL, total bilirubin; tCHOL, total cholesterol; TG, triacylglycerols; TyG, triglyceride-to-glucose index; UA, uric acid; WBC, leukocyte count.
